# On Peptides and Altered Peptide Ligands: From Origin, Mode of Action and Design to Clinical Application (Immunotherapy)

**DOI:** 10.1159/000448756

**Published:** 2016-09-20

**Authors:** Martín Candia, Bernhard Kratzer, Winfried F. Pickl

**Affiliations:** a Institute of Immunology, Center for Pathophysiology, Infectiology and Immunology, Medical University of Vienna, Vienna, Austria; b Christian Doppler Laboratory for Immunomodulation, Vienna, Austria

**Keywords:** Altered peptide ligand, Peptides, T cells, T cell epitopes, T cell activation, T cell responses, Immunotherapy

## Abstract

T lymphocytes equipped with clonotypic T cell antigen receptors (TCR) recognize immunogenic peptides only when presented in the context of their own major histocompatibility complex (MHC) molecules. Peptide loading to MHC molecules occurs in intracellular compartments (ER for class I and MIIC for class II molecules) and relies on the interaction of the respective peptides and peptide binding pockets on MHC molecules. Those peptide residues not engaged in MHC binding point towards the TCR screening for possible peptide MHC complex binding partners. Natural or intentional modification of both MHC binding registers and TCR interacting residues of peptides – leading to the formation of altered peptide ligands (APLs) – might alter the way peptides interact with TCRs and hence influence subsequent T cell activation events, and consequently T cell effector functions. This review article summarizes how APLs were detected and first described, current concepts of how APLs modify T cellular signaling, which biological mechanisms might force the generation of APLs in vivo, and how peptides and APLs might be used for the benefit of patients suffering from allergic or autoimmune diseases.

## Altered Peptide Ligands: General Considerations

In the past, a number of studies showed that CD4+ T helper cell activation cannot be regarded as a simple on-off phenomenon. In fact, T cell recognition is degenerate, i.e. single T cells can recognize a large collection of ligands. Consequently, T cellular responses represent a continuum of intensities and thus qualities which are the consequence of differences caused by amino acid substitutions in both major histocompatibility complex (MHC) molecules and antigenic peptides, i.e. T cell antigen receptor (TCR) ligands.

Altered peptide ligands (APLs) were first introduced into the field of antigen presentation by Evavold and Allen [1] in 1991 through study of the relationship between proliferation and cytokine production of T cell clones. In order to circumvent the problem of selection of T cell clones by their in vitro proliferative responses, they systematically replaced single amino acids in an immunogenic peptide and tested them for their T cell activation capabilities. Initially, studies centered on murine Th1 and Th2 clones with specificity for a peptide from the minor β^d^ chain of murine hemoglobin (Hb) [1, 2] (table 1). Of note, within the collection of peptides that still bound to MHC molecules, they identified one APL that modified the peptide-MHC complex (pMHC) in such a way that cytokine production became dissociated from proliferation [1]. In fact, the clones stimulated with this altered peptide did not proliferate but were still able to produce IL-4 at levels similar to those found when T cell clones were stimulated with the cognate peptide. The identified APL was also able to indirectly stimulate B cell activation and function, i.e. B cellular proliferation and antibody production, by fostering T cell-B cell cooperation.

These studies were promptly extended by different research groups to T cell clones with other specificities, such as influenza hemagglutinin, tetanus toxoid and ovalbumin (OVA) [3–6] and also included autoreactive T cells such as those which are frequently found in major human autoimmune diseases. Along those lines, the responses to APLs of myelin basic protein (MBP) and mycobacterial 65-kD heat-shock protein-specific T cells were analyzed and the possibilities of creating novel forms of peptide-based therapies for experimental allergic encephalomyelitis and adjuvant arthritis were evaluated [7, 8].

Evavold et al. [9] coined the term ‘APL’ to better describe antigen-derived peptides bearing single amino acid substitutions that stimulate some, but not all, T cell functions. With APL they were referring to those analogues of the wild-type, immunodominant peptides, in which distinct TCR contact residues had been structurally modified by usually conservative single amino acid substitutions [9]. Given the fast expansion of the research field, it did not take long until it was evident that not all the APLs generated and studied elicited the same responses in T cells. In fact, it turned out that APLs encompass a collection of antagonists, partial agonist as well as putative super-agonists of the immunodominant wild-type peptide [10], while still others without any clearly identifiable activity were revealed.

Partial agonists are incapable of inducing T cell proliferation; however, they can elicit some other features typically associated with T cell activation, such as cytokine release or cytolysis. Particularly for Th2 cells it has been shown that partial agonists are able to dissociate cytokine production and T cell-B cell collaboration from proliferation. Of particular importance in that context, partial agonists were identified, which were able to shift the phenotype of Th cells especially upon secondary stimulation. In studies conducted by Bottomly and coworkers [11], APLs for human collagen IV were found to induce a Th2 instead of the Th1 response, which is triggered by default upon encounter with the wild-type, agonist peptide. Similarly, in Th1 cells, APLs for a pigeon cytochrome C peptide (PCC_88–104_) and a moth cytochrome C peptide (MCC_88–103_) were shown to stimulate cytolytic activity, upregulate IL-2 surface receptor levels and increase cellular volume all in the absence of proliferation or cytokine production [12, 13].

In contrast to partial agonists, it was initially thought that complete antagonists are not delivering any identifiable signals to the T cells by themselves but that they are capable of significantly decreasing T cell proliferation, in a dose-dependent manner, especially when offered to antigen-presenting cells (APCs) simultaneously with the agonist, i.e. in the form of a competitive inhibitor [9]. However, evidence for antagonist-driven active inhibition has accumulated in subsequent studies performed with dual receptor-expressing T cells [14–17], with several studies demonstrating that antagonism induces negative feedback signaling via the phosphatase SHP-1 [18, 19]. Ostrov et al. [5] have shown that such functional differences are sensed by the clonotypic TCR, suggesting that a single peptide can stimulate one T cell clone while antagonizing another. All these studies pointed at differential TCR signaling and thus contributed to the better understanding of the T cell activation processes that was until then considered to represent an all-or-none phenomenon [20].

## Mechanism of Action of Altered Peptide Ligands

The mechanisms that account for these differential effects have been thoroughly analyzed in the past but are not yet fully clear as of today. The first model proposed for the mode of action of APLs was rather simplistic and entertained a mechanism in which only one of the signaling modules of the CD3 complex (ζζ vs. εγ and εδ) was activated upon TCR engagement when an APL was presented to the respective TCR. This was very much in contrast to agonist presentation, where full activation of all modules was observed [21]. Nevertheless, once detailed studies regarding early activation events and patterns of phosphorylation of molecules such as ZAP70 were performed, it became obvious that the signaling events comprised a higher complexity. In fact, Allen and coworkers [22] were able to demonstrate that when stimulating Th1 clones specific for Hb with anergy-inducing APLs, the pattern of phosphorylation induced by the phosphotyrosine kinases involved was unique, differing from that obtained by stimulation with the wild-type Hb peptide. In particular, the ratio between the tyrosine-phosphorylated and nonphosphorylated forms of the TCR ζ-chain was always significantly lower when using the APL for stimulation. Furthermore, the association of ZAP70 with the ζ-chain and its phosphorylation were also affected, with the phosphorylated form of the ZAP70 protein being almost absent. This clearly indicated that the subtle changes in the amino acid sequence of the stimulating peptide were unerringly translated into differential TCR signaling events as mirrored by altered engagement of downstream signaling proteins. In the latter experiments, McConnell and coworkers [23] also confirmed their initial findings by applying an APL of moth cytochrome C and showing that acid release, Ca^2+^-fluxing and T cell proliferation were hierarchical processes all susceptible to alterations by stimulation with the APLs. In this study, acid release measurements performed on a microphysiometer were used together with calcium fluxing experiments as complementary assays for identifying early events during T cell activation [24]. Moreover, other proteins within the TCR signaling pathway but clearly downstream of the ζ-chain were also shown to be differentially activated when T cells were activated by APLs. Amongst them is LnK, a signal transduction protein linking TCR signaling to PLCγ1, and PLCγ1 itself, therefore providing a link between the alteration of the early phosphorylation events and the subsequent alterations observed with regards to the degree of Ca^2+^ mobilization and fluxing [25]. Furthermore, differential activation kinetics might also play a decisive role in that context [25–29]. By the same token, accumulating evidence for differential signaling for antagonists has been revealed more recently with activation of SHP-1 taking center stage [18, 19]. Along those lines, Ding and Shevach [30] explored whether differential signaling induced by APLs could be overcome by forced engagement of costimulatory molecules on T cells such as CD28. In this study it was found that proliferation and IL-2 production could be clearly rescued by CD28 ligation; however, CD40L and IL-12Rβ2 chain expression were found to strictly rely on pMHC signaling with the APL applied revealing complete deficiency in providing the full-blown signal usually provided by the wild-type agonist [30]. In contrast, others rather found quantitative but not qualitative differences with regard to CD40L T cell expression imposed by APLs [31]. In addition, evidence for APL-induced modulation of the migratory behavior of lymph node and splenic T lymphocytes, by significantly reducing their adhesiveness for E- and P-selectins, has been reported in an experimental model of myasthenia gravis (MG) [32].

## APL Design

Initially, APLs were designed by introducing single amino acid substitutions into immunogenic peptides at the sites expected to point to and to interact with the TCR engaging the pMHC complex, as determined by X-ray crystallography. This encompassed alanine scanning of residues of interest [33, 34] followed by more or less conservative substitutions regarding the size and polarity of the most promising ones [25, 35, 36]. Alternatively, conservative substitutions would span all possible residues without a first alanine-scanning step [1, 37]. These approaches were used to generate the first APLs designed, such as the Hb-derived p.E73D designed by Evavold and Allen [1] or the MCC-derived p.K99R designed by Bottomly and coworkers [25], which induced cytokine production without T cell proliferation in the respective specific T cell clones.

A somewhat different algorithm focused on exclusively altering those peptide residues binding to MHC pockets to modify their affinity for the respective MHC molecules. This approach has been applied for peptides derived from tumor- or viral-antigens since a direct correlation between peptide-MHC binding affinity and immunogenicity could already be inferred early on [38–42]. Along those lines, Parkhurst et al. [42] designed a panel of melanoma gp100-derived APLs based on motifs derived from a collection of peptides isolated from HLA-A2 molecules. Several of these APLs, in particular p.T2M, displayed increased in vitro and in vivo immunogenicity. Furthermore, X-ray crystallography and computational modeling confirmed that the increased immunogenicity was indeed mediated by higher stability of the pMHC complex [43]. Ultimately, this also caused significant alterations of the three-dimensional structure between TCR and the pMHC complex [44], indicating altered TCR recognition.

Other approaches taken include the modification of residues adjacent to those directly in the MHC groove both at the carboxyl- and amino-terminal end [45, 46]. These are usually based on observations of natural variations of the particular peptide indicating that modifications of such residues have a direct impact on the peptide-MHC binding affinity.

More recent studies took another important step towards the rational design of APLs by applying straight-forward solutions to their in silico design and optimization. By using structural information of common HLA class II molecules, Chen et al. [47] optimized gp100-derived peptides by substituting MHC anchor residues for increased binding to HLA-DR4, hoping to thereby enhance the activity and action of antimelanoma-specific T cells. As planned, the in silico optimized APL revealed increased HLA-DR4 binding as tested in classical pulse/chase experiments using ^125^I-radiolabeled probes. However, unlike the results with a gp100-specific T cell clone showing superior responses to p.Q56A than to the wild-type peptide, the p.Q56A APL was not consistently superior to the wild-type peptide when reanalyzed by polyclonal T cells derived from 6 patients who had undergone vaccination with the wild-type peptide [47]. Thus, while structural considerations provide facile means to optimize peptide binding strength, the functional consequences of peptide alterations cannot be fully predicted but have to be meticulously analyzed by functional means in polyclonal T cell populations.

## Influence of Altered Peptide Ligands on T Cell Development and Polarization

The differential behavior (partial antagonism and antagonism) of APLs together with the notion that they were capable of altering TCR signal strength when compared to the agonist (see above) rendered APLs as perfect tools with which to study T cell selection in the thymus. In fact, independent studies by Jameson et al. [48], Ashton-Rickardt et al. [49], and Hogquist et al. [6], all using fetal thymus organ cultures and testing the effects of APLs helped to demonstrate that during thymic selection pMHC complexes, but not mere MHC molecules alone, are the crucial selection structures for the developing thymocytes. Moreover, these studies also demonstrated that peptides do not only stabilize MHC molecules, but provide important specificity for the positive selection process [6, 48, 49]. Furthermore, just two years before the AIRE gene was described, the use of APLs helped Allen and coworkers [50] to support their model of the two avidity thresholds during positive and negative selection. This model suggested that in the thymus, for each TCR, there could be several ‘lookalike’ peptides that would be able to interact with that TCR, all of them with slightly different affinities. In this way, a ‘window’ of affinity defined by a lower and upper affinity threshold would be set by these peptides. T cells expressing TCR with affinities outside of this window would be either positively or negatively selected. Along those lines, Allen and coworkers [50] used a transgenic mouse model based on a T cell clone specific for the β-chain of murine Hb. These mice expressed the corresponding transgenic TCR β-chain paired with the endogenous α-chain. This resulted in a unique system in which the clones isolated were reactive against an APL of the Hb^b^_64–76_ peptide displaying Ser-69, while being antagonized by the original, wild-type peptide. In this model, the APL became an agonist while the original agonist turned into an antagonist. With this particular setup, it was possible to assess how the endogenous expression of Hb altered the selection of the TCR transgenic T cells in the thymus. Importantly, they found that the T cell populations with high or too low avidity for Hb^b^_64–76_ were absent in transgenic mice, which was in clear contrast to mice treated with the wild-type control peptide [50]. Consequently, the ‘window of avidity model’ was confirmed since the obtained results were in clear concordance with the findings that low affinity interactions favored positive selection while high-affinity interactions favored negative selection [6, 49]. More recent studies have shown that, in fact, recent thymic emigrants (RTE) respond with increased expansion upon stimulation with low-affinity APL, which is accompanied by increased levels of VLA-4 expression leading to improved migration to inflamed organs [51]. This was reflected by faster and more intensive ERK phosphorylation in RTE compared to mature T cells. The excellent reactivity with the APLs studied might indicate that RTE respond to a broader range of peptides and, during infection, might give rise to an early wave of short-lived effector cells [51].

As mentioned above, APLs were not only used in studies dealing with various aspects of T cell maturation but with great enthusiasm also in those centering on T cellular polarization in which they were, in fact, identified to induce differential polarization patterns (see above). This certainly belongs to the most interesting features of APLs because it identified them as attractive candidates for reshaping the function of pathognomonic T cells. Consequently, APLs were meticulously analyzed for their therapeutic potential in otherwise difficult-to-treat autoimmune diseases, such as multiple sclerosis (MS), rheumatoid arthritis (RA) and others.

The first studies demonstrating a polarizing potential of APLs were, however, conducted by the group of Bottomly and colleagues [34] in different model systems. Initially, they studied naïve CD4+ T cells isolated from the lymph nodes of transgenic mice expressing a TCR specific for the MCC peptide 88–103 bound to I-E^k^ or I-E^b^. Naïve T cells were primed with a constant amount of 5 μg/ml of different APLs thereof. After a resting phase of 48 h, the restimulation of these cultures with wild-type peptide alone led to high production of IL-4 by these T cells primed with APLs, as compared to those primed with the agonist peptide, which almost exclusively produced IFN-γ. In particular, APL p.K99R was shown to induce high levels of IL-4 and this property was again dependent on the peptide dose. Interestingly, very low doses (0.05–0.5 μg/ml) induced predominantly IFN-γ production while intermediate doses (5–50 μg/ml) led to high-level production of IL-4 and high doses (500 μg/ml) again induced a predominance of IFN-γ production.

These results were corroborated by in vivo studies using I-A^b^ mouse strains, which developed a Th2 response upon priming with a huColIV 12-mer peptide, or those expressing the I-A^s^ allele, which developed a Th1 response when stimulated with the same agonist [11]. For the first strain, two APLs were identified, i.e. α2Glu(5) and α2Ala(11) (p.P679E and p.P685A, respectively), both of which completely reversed the Th1 into Th2 phenotype as assessed by the determination of mRNA expression levels for IL-4 and IFN-γ in CD4+ T cells isolated from lymph nodes. Similar results were obtained with the second strain of mice, in which the APL α2Ala(10) (p.G678A) induced IFN-γ in the absence of IL-4 expression, exactly opposite to what was observed for the agonist [11]. Additional information gained from the collagen-induced arthritis model centered on APLs generated from human heat-shock protein 60 (HSP60) [52, 53]. Evidence was provided that APL1 induces CD4+CD25+ Treg but drives CD4+CD25+ activated effector cells into apoptosis [52]. Another HSP60-derived APL, APL2, reportedly induced IL-10 in PBMCs derived from idiopathic arthritis patients [53].

Another interesting model represents experimental autoimmune MG in which autoantibodies become directed against AChR (acetylcholine receptors) of skeletal muscles which cause muscular weakness and excessive fatigue. Two peptides comprising sequences of the human AChR (p195–212 and p259–271) have been shown to stimulate peripheral blood (PB) lymphocytes of patients with MG. A dual altered peptide ligand combining the two single amino acid analogs of p195–212 and p259–271 were shown to inhibit the activation of lymphocytes by myasthenogenic peptides and to ameliorate the clinical course of experimental autoimmune MG [32, 54, 55]. Active suppression by the dual APL was mediated by modulation of cytokine secretion, leading to increased TGF-β and IL-10, and decreased IFN-γ and IL-2 secretion. Significantly, the suppressive phenotype could be adoptively transferred by cells of treated animals [55]. In addition, the same group of authors have shown that the dual APLs also induce CD4+CD25+ [56] as well as CD8+CD28− [57] T cells. The CD4+CD25+ T cells, which upregulate a 50-kDa ERK-like protein upon dual APL stimulation [58], seem to maximize their regulatory function by coexpressing CTLA-4 and – along with the secretion of TGF-β – induce apoptosis of effector T cells [59].

## Naturally Occurring Protein Modifications Leading to APL Formation

Given that most APLs differ from their agonists only in one single amino acid, it is not difficult to envision that ‘natural mechanisms’ exist by which such derivate peptides could be generated in biological systems, including the human body. These might encompass simple transcriptional or translational errors introduced by the replication machinery of the respective cells involved as well as posttranslational modifications of proteins produced within the body. Moreover, this also includes substances introduced into the body but derived from environmental sources, such as inhaled proteins, dietary proteins, or proteins derived from microorganisms in the course of an infection or simply from the microbiome. As a matter of fact, single mutations found in viral but also parasitic genomes, e.g. those in HIV [60, 61] and hepatitis B and C virus [62–64] or plasmodium [65], and resulting in the production of such APLs were indeed described. These mutations were proposed as passive mechanisms by which microorganisms might counteract the T cellular immune response by inducing anergy in pre-existing human T cells that are specific for the original/wild-type epitopes.

Compatible with these findings, several ‘naturally occurring’ APLs have been identified and linked to the establishment and/or development of distinct autoimmune diseases, including celiac disease (CD) and RA; however, such APLs are not the result of bona fide mutations but are rather the consequence of posttranslational modifications changing the chemical composition of proteins and/or immunodominant peptides generated thereof. Post-translational modifications have been shown to encompass, among other mechanisms, deamidation of asparagine to aspartic acid or iso-aspartic acid, as suggested by McAdam et al. [66] and Mamula et al. [67]. Moreover, Hill et al. [68] have shown related phenomena in RA in which deamination of arginine leads to the formation of citrullinated proteins, which in turn leads to increased peptide:MHC affinity and thus to the enhanced activation of CD4+ T cells in HLA-DR4 IE transgenic mice. Critical events seem to be those in which the affected arginine, which becomes modified to citrulline, engages the positively charged P4 anchoring pocket. Citrullination modifies the positively charged imino side chain group to an uncharged carbonyl group and thereby dramatically increases the affinity of the modified peptide for binding to the HLA-DRB1*0401 allele, known to be strongly associated with RA. This explains how posttranslational peptide modification could shape the immune response to otherwise innocuous antigens/peptides and also illustrates how the additional T cell help might drive (auto)antibody production. This assumption fits well with the strong correlation between the appearance of anti-citrulline antibodies and certain HLA alleles highly enriched within the collective of RA patients, which also clearly argues for the important role of T cell help for anti-citrulline antibody production and their contribution to mounting high titers of anti-citrulline antibodies [69]. Notably, citrullination is not a phenomenon exclusively restricted to the formation of arthritogenic peptides as it has also been described to occur in proteins typically associated with other autoimmune diseases. It also affects MBP-derived peptides and might thus also be of relevance for diseases of the central nervous system, such as MS [70].

What are the triggers for such posttranslational peptide modifications? Of relevance in this context are the findings by Ireland et al. [71] revealing that protein immunization with hen egg white lysozyme (HEL) leads to the presentation of citrullinated HEL peptides, which can be recognized with specific T cell hybridomas. In their studies, citrullination of proteins resulted from the action of peptidyl arginine deiminases (PAD), a group of enzymes overexpressed in inflammatory cells such as neutrophils, monocytes and macrophages [72]. The authors hypothesized that the massive inflammatory response induced by supplementation of the model antigen HEL with complete Freund’s adjuvants might be the driver for the preferential formation of citrullinated, immunodominant peptides. Crucial for the further understanding of the generation, presentation and recognition of citrullinated peptides will be the better characterization of the regulators of the PAD. In a transgenic system of HEL overpresentation, in which HEL was conjugated to the TM region of L^d^ under the IE promoter, the authors showed that apart from the native, immunodominant HEL_48–62_ peptide, also the citrullinated version of the peptide, i.e. HEL_48–62_ cit61, became copresented. As a read-out they used T cell clones specific for either of the peptide forms. In marked contrast to the double-edged presentation by DC, B cells only presented the unmodified, native peptides, indicating that different APCs might considerably differ in their capability to generate and thus present citrullinated peptides, which might be a function of PAD activity. Notably, B cell starvation, which represents a strong inducer of autophagy, also leads to the presentation of citrullinated peptides in this cell type [73].

Deamidation processes, along with the formation of immunogenic peptides, are also typical features of another autoimmune disease, i.e. CD. CD is an enteropathy that precipitates when genetically predisposed individuals become exposed to the cereal storage protein gluten, derived from barley, rye and wheat, or to its component gliadin. Predisposed individuals carry either a HLA-DQ2 or a HLA-DQ8 haplotype [74–77]. The great majority of patients with CD develop IgA autoantibodies directed against the extracellular matrix-associated enzyme tissue transglutaminase 2 (TG2) [78, 79]. TG2 can bind gliadin peptides and link these peptides covalently to transglutaminase by the formation of iso-peptide bonds between Q and K residues [80]. Notably, TG2 activity is increased in the affected gut tissues of CD patients [81] and T cells specific for gliadin can be isolated from the lamina propria of patients [82, 83]. In this way, gliadin transforms from an innocuous to an immunogenic protein, eventually resulting in a Th1 T cell response against the gliadin components.

Importantly, TG2 has also strong deamidation activity converting glutamine residues into glutamic acid, thus converting polar into negatively charged side chains. Fully compatible with this finding, T cells isolated from lamina propria samples of patients [84, 85] recognize deamidated gliadin peptides with high specificity. Furthermore, deamidation renders these peptides more antigenic than their nonmodified analogues [84, 85]. In fact, deamidation is selective for proline/glutamine-enriched sequences and a wide range of gluten-derived peptides containing such sequences that are targeted by TG2 have been identified and are able to activate responses by lamina propria-resident T cells isolated from patients [86–88].

Of particular interest in that context, structural analyses of the HLA-DQ2 and HLA-DQ8 molecules have shown that both molecules contain anchoring pockets that preferentially bind negatively charged residues. These are P4, P6 and P7 for HLA-DQ2; and P1 and P9 in the case of HLA-DQ8 [89, 90]. Consequently, the post-translational modification of gliadins by TG2 enhances the affinity of the respective peptides derived from them for binding to these HLA molecules [84, 88, 91].

In the case of patients with an HLA-DQ2 background, α-gliadin has been found to be the major antigen. Two independent groups have demonstrated with PB- [92] or gut biopsy-derived T cells [93] from CD patients that only one dominant α-gliadin T cell epitope exists, which comprises amino acid residues 57–73, containing a single deamidation site at residue 65 [92, 93]. Of particular interest, an extended 33-mer peptide spanning residues 57–88 and containing six partially overlapping sequences from three T cell epitopes has proven to be highly immunogenic after deamidation by TG2 [87, 94, 95]. This 33-mer peptide is currently being studied for the development of blocking analogue peptides (pharmacological antagonists) suitable for patients with a HLA-DQ2 haplotype. Although the 33-mer peptide contains several epitopes, it binds with a 1:1 stoichiometry to the respective MHC molecules, suggesting that possible secondary structures outside the HLA/TCR groove may play an important role for the further stabilization of its interaction [96]. Based on this sequence, two promising peptides have been derived. Both harbor a double leucine to lysine substitution at positions 11 and 18, the second peptide represents a homodimeric form of the first peptide. Using in vitro T cell activation assays, both peptides have shown strong blocking activity for the 33-mer peptide as well as for several α-gliadin and γ-gliadin peptides [96].

Apart from deimidation, deamidation and citrullination events, several additional posttranslational changes of protein and peptide sequences isolated from MHC molecules have been reported, including phosphorylation and nitration of tyrosine residues [97, 98], the addition of saccharides and also of small chemical groups functioning as haptens, e.g. trinitrophenyl residues [99]. The drivers for many posttranslational modifications might be activated macrophages, dendritic cells or B cells providing nitric oxide and reactive oxygen species along with the formation of other strong cell stress-inducing molecules. In fact, infections might promote autoimmunity by inducing neo-epitopes to which autoreactive T cells, which are present in the periphery, start to react. The extent and quality of macrophage and dendritic cell activation might play a critical role in that context. Modifications might take place in several antigen-presenting compartments, including late vesicular compartments but also recycling early vesicles [100].

Apart from the above-described autoimmune diseases, hapten-specific T cells also play a major role during the development of metal and drug hypersensitivities [101–103]. Significantly, it has been shown that the undesired reactivity of hapten-specific T cells can be altered by simply modifying the culprit hapten, thereby inducing T cell anergy in the reactive T cell clones. Such ‘altered hapten-peptide ligands’ have been investigated by Preckel et al. [104, 105] showing that OVA-derived SIINFEKL peptides modified with DNP instead of TNP induce a reversible, anergic state in SIINFEKL-TNP-specific T cells. The findings with altered hapten-peptide ligands add to the overall concept that chemical modification of an immunodominant peptide, especially when the relevant TCR contact site(s) are concerned, might induce an altered T cell response when presented to and recognized by the specific TCR. These experiments demonstrate that, apart from variations in the primary amino acid sequence of immunodominant peptides, also secondary, chemical modifications, e.g. introduced by reactive groups or compounds, might impact on peptide recognition and in turn influence T cell function. Furthermore, it also shows potential ways of how to influence the reactivity of drug-specific or autoreactive T cells, as those present in RA (see above) [68] and drug hypersensitivity [103].

## Type B Peptide Binding and Type B T Cells as a Form of Alternative Peptide Presentation and Recognition

Yet another major mechanism of how alternative peptides might become presented and induce altered T cell responses has been meticulously described by Unanue and coworkers [106–109]. Most interestingly, they have identified two sets of T cells, which differ in their recognition of the same immunodominant peptide segments of HEL, amino acid residues 48–61. Type A T cells are conventional T cells, which recognize the MHC-bound peptide resulting from the processing of the conformationally intact HEL protein in the intracellular processing compartment. Such T cells also recognize the immunodominant peptide when offered to APCs as exogenous peptide and bound to cell surface-expressed MHC, indicating that these peptides do not necessarily require intracellular processing to become properly recognized by specific T cells. This is very much in contrast to type B T cells, which exclusively recognize peptides bound exogenously but not those derived from intracellular processing of the whole protein [110]. Unanue and colleagues [106, 109] suggested that the type B state of pMHCs might result from a unique/alternative conformation of the involved peptides, which can only be engaged when destabilized exogenous proteins/peptides bind to surface-expressed or early endosomal-resident MHC class II proteins. The accidental generation of type B pMHCs in the periphery along with the activation of type B T cells could be an important trigger for autoimmune reactions and might be the motor for the expansion of type B T cells. According to this hypothesis, inflammatory conditions in the periphery might create a milieu, which promotes cell death and extracellular protein destabilization and degradation, favoring the formation of a pool of proteins/peptides amenable to take on ‘type B configuration’ upon binding to cell surface-expressed MHC molecules. Under physiological conditions, no such extracellular protein degradation environment is present, which might explain why type B-reactive T cells could escape negative selection in the thymus, can populate the periphery and assume an autoimmune phenotype when entering into chronically inflamed tissues, for example. Biochemically, discrimination between proteins/peptides bound to surface-expressed MHC molecules versus MHC molecules expressed in the MIIC is possible and can be assessed by their stability upon exposure to the nonionic detergent sodium lauryl sulfate (SDS) [111, 112]. It is well known that proteins/peptides bound to surface-expressed MHC molecules generally give rise to SDS-unstable pMHCs and are the resultant of partially folded molecules/peptides binding to receptive MHC class II molecules [113]. This is in contrast to SDS-stable complexes, which result from intracellular processing of whole proteins and give rise to long-lived pMHCs. Conformational flexibility and/or destabilization of a protein/peptide when associating with a receptive, surface-expressed MHC molecule might thus favor alternative modes of peptide binding, possibly taking advantage of different registers for association with MHC molecules when compared to the immunodominant peptides derived from proteins processed intracellularly in the MIIC. This mode of peptide binding is independent of the classical protein-processing machinery and can thus also be observed with chemically fixed APCs. That type B T cell reactivity is the mere result of posttranslational protein modification (see above) has been heavily disputed by Unanue and coworkers [114].

## Altered Peptide Binding Influenced by Cofactors Such as MHC Loading Enhancers

The amounts of immunogenic peptides presented to antigen-specific T cells upon primary and secondary encounter has a strong impact on the subsequent functional output of the respective T cells. Of interest in that respect is the observation that the degree of peptide loading can be modulated by substances impacting on the ‘receptivity’ of cell surface-expressed MHC molecules. Under steady-state conditions in professional APCs, the efficient loading of MHC class II molecules with peptides is accomplished by the chaperone molecule DM [115]. This process takes place in the acidic environments of the MHC class II peptide loading compartments (MIIC) in APCs [116, 117]. Notably, exogenously administered peptides applied with the intention to modulate immune responses face the intrinsic problem that they are becoming rapidly degraded by proteases present in almost all bodily fluids. The in vivo half-life of immunogenic peptides can, however, be prolonged by substances catalyzing and thus accelerating DM-independent peptide loading onto cell surface-expressed MHC class II molecules or those residing in early endosomal compartments [118]. In the past, several groups of small molecules catalyzing peptide exchange in a DM-independent manner, and thus acting as ‘MHC loading enhancers’ (MLE), have been identified. This collection includes simple alcohols [118], short linear peptides of 2–7 amino acids in length [119], circular peptides [120], adamantyl compounds [121], inorganic metal complexes and other small-molecule enhancers [122]. What are the mechanistic principles of these substances? MLE are believed to interfere with and to interrupt the hydrogen bonds, which are formed between peptides and MHC class II molecules and thereby facilitate peptide exchange [118]. By that mechanism MLE would also turn already empty MHC molecules from a peptide-nonreceptive into a peptide-receptive state, and by doing so they facilitate their loading with peptides present only in the extracellular environment. This not only enhances the reactivity against already immunogenic peptides by increasing the number of pMHC complexes, but might also induce immune responses against antigens, which would otherwise remain ignored by the immune system. Since several drugs, such as the antiviral and anti-Parkinsonian drug rimantadine [123, 124], as well as the antidiabetics saxagliptin [125] and vildagliptin [126], are in fact based on the adamantan-like cage structure, it can be envisioned that they themselves or their metabolites might impact on the repertoire of peptides presented in individuals taking the respective drug. Other MLE, such as those based on di-peptides, depend for their generation on the presence and activity of dipeptidyl peptidases, which in fact also have prominent representatives in the immune system such as dipeptidyl peptidase IV, also called CD26 [127, 128], a molecule highly expressed on T lymphocytes [129]. The generation and repertoire of dipeptides might, apart from pharmacological modulators and the activity of peptidases, also depend on the availability of appropriate substrates, i.e. those ingested as nutrients or those that are a part of microorganisms. That MLE-catalyzed peptide exchange indeed leads to enhanced biological responses also in vivo has been very convincingly shown in a report by Dickhaut et al. [130] demonstrating superior tumor-specific immune responses against NY-ESO 1 protein upon s.c. application of NY-ESO peptide in the presence of adamantyl ethanol. Similar improvements have been shown with the whole encephalitogenic MBP protein [122, 130, 131]. Thus, enhanced loading of peptides increasing the presentation by APC might per se represent a mechanism to alter TCR signaling and thus modify subsequent T cellular responses.

## Allergen-Derived Altered Peptide Ligands and Their Function

Evidence for the natural occurrence of APLs of major allergens has been described in the past. In the field of allergies, the phenomenon of cross-reactivity has been well described not only for B cell epitopes, but also for T cell epitopes [132–139] in particular between major pollen and major food allergens [140–143]. A thoroughly studied system is that of Bet v 1, the major birch *(Betula verrucosa)* pollen allergen. Jahn-Schmid et al. [132, 144–146] identified Bet v 1_142–156_ as the immunodominant T cell epitope. Moreover, they have shown that Bet v 1_142–156_-reactive TCL and TCC also become activated in response to homologous peptides derived from major food sources, such as apple (Mal d 1), cherry (Pru av 1), carrot (Dau c 1), celery (Api g 1) and hazelnut (Cor a 1). The homology between Bet v 1_142–156_ and the cross-reactive peptides ranges between 73 and 80%, which indicates differences in 5–8 amino acids, with 33–66% of the amino acid exchanges being conservative. Some of these T cell epitopes become exposed upon digestion and remain fairly stable, e.g. Api g 1, Mal d 1 and Cor a 1, enabling them to activate a distinct fraction of Bet v 1-specific TCC [147]. Together, these are excellent examples for the occurrence of ‘natural APLs’ of an immunodominant aeroallergen. While primary stimulation with these peptides did not provide any evidence for differential T cell activation [132], it remains to be shown whether preexposure to the respective food-derived peptides would alter T cell function upon reencounter with the immunodominant Bet v 1 epitope.

Apart from these naturally occurring APLs, manufacture of altered peptide ligands of a restricted number of major allergens has been described in the past. The first in the series was an APL described for a T cell clone specific for the Japanese cedar pollen allergen Cry j 1 [148]. This clone recognized the Cry j 1_335–346_ peptide in a HLA-DRA/HLA-DRB3*0301-restricted manner (table 2). Out of a series of analogue peptides generated by the nonconservative exchange of single amino acids, five peptides were identified by the authors, which lost their T cell-activating capacity. However, peptide binding assays identified 2 out of these 5 peptides (^338^Leu and ^341^Asn) as having also lost their MHC binding capacity. Moreover, residues 337 and 342 were identified to be important for interactions with the TCR and/or the HLA molecules, respectively. Analogue peptides inducing no proliferation were further analyzed to determine whether they functioned as TCR antagonists. Peptides with the substitutions p.T339G and p.T339Q induced 70% less T cell proliferation and no IL-4, IL-2 or IFN-γ secretion when compared to wild-type peptides, which identified them as potential antagonists. Of note, another peptide with the substitution p.T339V induced significantly more IFN-γ, although proliferation rates were similar to the wild-type peptide. Thus residue ^339^Thr was identified as a ‘hot spot’ for altered T cell recognition within the Japanese cedar pollen allergen Cry j 1_335–346_ peptide, which led to altered T cell responses in this model.

The group of Yssel and coworkers [149] analyzed in detail the response of Der p 1-specific T cell clones against modified synthetic peptides directed against the house dust mite major allergen peptides Der p 1_94–104_ and Der p 1_171–182_. Using three Th2 T cell clones and autologous EBV-LCL clones, they identified in an Ala/Gly-scan those peptide substitutions that failed to induce T cell proliferation when compared to the wild-type peptide. Notably, the lack of the proliferation-inducing capacity of these peptides was in every case also accompanied by the inability to induce cytokine production [149]. Thus, in contrast to the previous report, herein no dissociation between cytokine production and proliferation could be observed. Since the authors did not perform any binding assays to the respective HLA restriction elements with the APL under investigation, they could not really tell whether the observed lack of proliferation was due to a lack of HLA binding, a lack of TCR engagement, or both. Nevertheless, they analyzed whether the identified peptides would antagonize the action of the wild-type peptide when coincubated at 10- to 100-fold excess. Indeed, three out of the four nonstimulatory Der p 1_94–104_ peptides and two out of the six Der p 1_171–182_ peptides antagonized proliferation of the T cell clones induced by the respective wild-type peptides. The in-depth analysis of amino acid residue 173 in the Der p 1_171–182_ peptide modified by alternative amino acid substitutions revealed peptides with varying antagonistic activities, ranging from 9 to 78%. Antagonism was dose dependent, with maximum inhibition detected at a 100-fold molar excess of the antagonist. Remarkably, the antagonists inhibited IL-2 and IFN-γ production more dramatically than IL-4 production. In addition, to the APL-induced inhibition of proliferation and cytokine production, coincubation with antagonistic peptides also inhibited CD154 (CD40L) neo-expression on the T cell clones as well as IgE production by cocultured B cells. In summary, Yssel and collaborators [149] have convincingly shown that the antagonistic Der p 1 peptides identified also block T cell-dependent class switch recombination and subsequent IgE production by B cells in vitro. They did, however, not find any indication that the antagonistic peptides would induce segregation of proliferation from cytokine production, as has been shown so convincingly for APLs in murine model systems [1, 37]. The direct in vivo application of such HDM-derived antagonists is certainly limited by the fact that house dust mite extracts consist of a considerable number of different major allergens [150–152], which are recognized by a whole collection of different HLA class II molecules. Thus, approaches using more than one APL for clinical therapy would certainly be required.

Antagonistic peptides have also been described for the major allergen of *Parietaria judaica*, Par j 1 [153]. Three T cell lines were used to identify the T cell stimulatory peptide Par j 1_47–65_. Substitutions with alanine or valine residues at amino acid positions of Par j 1_47–65_ which seemed unlikely to represent major anchor residues led to the identification of three peptides with a reduced T cell stimulatory capacity. All three peptides clearly bound to the whole range of HLA class II molecules (HLA-DR1, -DR4, -DR3) examined. In fact, affinity determination revealed that the altered peptides had a lower affinity for HLA-DR1 than the wild-type peptide. The fact that all three peptides identified, i.e. ^52^Val, ^57^Ala and ^60^Ala blocked the response of the T cell lines coincubated with either wild-type peptide or the entire Par j 1 protein, indicated that the peptides did not exert their inhibitory function by blockade of the HLA molecules, but rather by bona fide TCR antagonism. This interpretation was corroborated by the fact that the altered peptides were used in a concentration range that maximally represented a 2-fold excess over wild-type peptides [153]. As the authors put it, this made peptide competition for available HLA-DR molecules (HLA blockade) an unlikely explanation for the observed inhibition in their experiments [153]. De Palma et al. [153] pointed out that altered Par j 1_47–65_ peptides that downregulate specific T cell responses could eventually also lead to lower IgE production. The promiscuous binding behavior of the Par j 1_47–65_ peptide and its antagonists, which have been shown to bind to a number of common HLA class II alleles, makes such an approach plausible.

For the major bee venom allergen phospholipase A2 (PLA), an altered peptide ligand specifically inhibiting Th2 cytokine synthesis has been characterized [154]. In this study all residues within the immunodominant PLA_81–92_ peptide were substituted by alanine. While substitution of the C- and N-terminal amino acids of the PLA_81–92_ peptide did not have any influence on their T cell stimulatory capacity, similar substitutions at residues 84, 86, 88 and 89 completely abrogated the T cell stimulatory capacity of the respective altered peptides. Notably, alanine substitutions at the residues 82, 83, 85, 87 and 90 reduced proliferation of T cell clones under study. Moreover, cytokine secretion experiments showed that the pattern of IL-4 and IFN-γ production in response to the APLs directly correlated with the proliferation data, i.e. they were generally reduced. However, APL p.F82A stood out with respect to its clearly more prominent reduction of IL-4 than IFN-γ production. This effect of APL p.F82A was confirmed by investigating PBMCs from an allergic donor, in which APL p.F82A, in contrast to the wild-type peptide, did not induce any IL-4 production, while similar amounts of IFN-γ were induced by both peptides, corroborating the preferential decrease of the IL-4:IFN-γ ratio. Of note, the Th2 cytokine repressing nature of APL p.F82A also led to increased levels of IgG4 production in vitro. Binding experiments showed that APL p.F82A, in fact, bound with lower affinity to the restricting HLA-DP molecules. Additionally, the authors found that preincubation of the T cell clone with either the wild-type peptide or APL p.F82A for 16 h in the absence of APC induced a state of anergy in such treated T cells. In fact, subsequent reculture of the thoroughly washed T cells with autologous APC in the presence of wild-type peptide showed both suppressed cytokine production and proliferation. This might be the result of peptide presentation between T cells in the absence of IL-2, resulting in the secretion of high levels of IL-10 which might, in fact, synergize with the IL-2-depriving effect [155, 156]. Finally, biochemical experiments demonstrated reduced ZAP-70 tyrosine phosphorylation in peptide-treated T cells. The authors concluded that such PLA-specific APLs, which alter the pattern of secreted cytokines in restimulated T cell cultures, may improve the efficacy of specific immunotherapy, alone or in combination with the wild-type peptide or even as a component of a recombinant protein [154].

Two different APLs have been defined for the lipocalin Bos d 2 by single amino acid substitutions at position 135 (p.N135D) and 133 (p.Q133K) [157, 158]. These APLs have the peculiar characteristic that they stimulate Bos d 2_127–142_-specific T cell clones already at much lower concentrations than the wild-type peptide. This is in contrast to saturating peptide concentrations, at which both wild-type and modified peptides induced a similar degree of proliferation. This was also mirrored in experiments determining the degree of TCR modulation as a sign of T cell activation. APLs efficiently modulated the surface expression of allergen-specific TCR already at lower concentrations and to a larger extent when compared to the wild-type peptide. The differences observed in TCR downregulation experiments were mirrored by CD25 neo-expression and confirmed that compared to the wild-type peptide much lower concentrations of the APL were required to induce a similar degree of T cell activation. Using tetramers loaded with either wild-type peptide or APL, the authors confirmed that the TCR of the allergen-specific T cell clones have a higher affinity for the APL as compared to the tetramers loaded with wild-type peptide. Moreover, the IL-4/IFN-γ ratio was decreased when T cell clones were cultured with APL, revealing the induction of a Th2 to Th1 shift. This might be a reflection of the increased TCR signal brought about by the APL. Notably, the APL induced a higher degree of cell death during a 10-day observational period when compared to the wild-type peptide, which was compatible with previous observations. The authors concluded that the heteroclitic APL developed by them deviate the immune response towards Th1 and induce more T cells to undergo apoptosis or to enter a state of hyporesponsiveness when compared to the wild-type peptide.

A major drawback of these studies was, however, that none of the human-relevant allergens under investigation could be evaluated in a meaningful way in vivo due to the lack of humanized animal models with specificity for them. That APLs, in principle, can impact on allergic inflammation and airway hyperresponsiveness has recently been shown for the model allergen OVA [159]. OVA represents a frequently used model allergen in allergy research (although not human relevant) especially when it comes to test experimental airway hyperreactivity. In the report by Janssen et al. [159] several peptide analogues of the immunodominant T cell epitope of OVA_323–339_ have been generated and characterized. Among the 12 peptide analogues, two failed to stimulate while two partially stimulated OVA-specific DO11.10 T cells. Seven peptide analogues induced similar proliferation and cytokine production by DO11.10 T cells as the wild-type peptide. Notably, the peptide p.Q336A was identified as a superagonist, revealing both increased proliferation rates as well as enhanced IFN-γ and IL-2 production. Thus, p.Q336A induced a shift of the cytokine profile already during primary stimulation. When Th2-biased cell lines obtained after repeated (3×) stimulation with the wild-type peptide were restimulated with p.Q336A, only IFN-γ but no IL-4 and IL-5 was produced by them. This was in contrast to restimulation with the wild-type peptide, which consistently led to high IL-4 and IL-5 but low IFN-γ production. This clearly demonstrated that the superagonistic p.Q336A peptide not only induced a Th1-bias during primary stimulation, but that it is also able to modulate already polarized Th2 cells towards a Th1 phenotype. For the subsequent in vivo studies, the candidate peptides were incorporated into liposomes in order to improve their in vivo half-life, which is otherwise limited by proteases present in bodily fluids. In fact, therapy with liposome-encapsulated p.Q336A peptide analogues of OVA-sensitized mice revealed significantly reduced BAL eosinophilia levels upon rechallenge with OVA. Moreover, peptide therapy with the superagonist significantly reduced IL-4 and IL-5 production in lung-draining lymph node cells, while it did not have any impact on OVA-specific immunoglobulin levels. This study very clearly showed that administration of Th2-skewing peptides might aggravate while Th1-skewing peptides can ameliorate allergic diseases, at least in the experimental setting of OVA-induced hyperreactivity [159].

## Therapy with (Wild-Type) Peptides and APLs

The recognition of peptides displayed by APCs is crucial for the induction of primary T cell immune responses. In certain diseases, including IgE-associated immune reactions, the T cellular immune response, besides other factors, decides whether an individual will develop a protective (blocking antibodies, Treg cells) or a pathological (IgE-driven) immune response. To appropriately modulate the respective immune response into the desired, protective direction, peptide immunotherapy was introduced as a possible therapeutic modality. In peptide immunotherapy patients are treated with nonmodified, wild-type, immunodominant peptides [160]. The current hypothesis postulates that peptide immunotherapy influences the course of disease mainly by the induction of Tregs producing the immunosuppressive cytokine IL-10 [161]. Moldaver et al. [162] could even demonstrate that in polysensitized mice (OVA and house dust mite), house dust mite-specific peptide therapy ameliorates OVA-induced asthma and induces IL-10 in the absence of IL-35 induction. However, Briner et al. [163] earlier described reduced T cell proliferation and cytokine production and speculated about clonal deletion of the culprit allergen-specific T cells as the major mechanism by which peptide-induced tolerance is mediated. In fact, the reduction of allergen-specific T cells upon peptide immunotherapy was demonstrated by Campbell et al. [161] in 2009. Furthermore, the same authors observed linked epitope suppression, as T cells to the same and a related peptide showed reduced proliferation capacities along with reduction in the production of the Th2-associated cytokines IL-4 and IL-13. The findings were accompanied with an increase in CD4+IL-10+ but not CD4+FoxP3+ T cells as well as with an increase in IL-10 but not in active TGF-β levels in BAL and lung tissue homogenates [161].

Previous studies have shown that T cell stimulation with supraoptimal doses of high-affinity antigen induces T cell tolerance through the establishment of a state of hyporesponsiveness and/or cell death [164–168]. The observed outcome may also depend on the chosen immunization route, as for example intranasal delivery may lead to mucosal tolerance induction [169].

Notably, IL-10-dependent immunoregulation induced by peptide immunotherapy was observed in other murine models of allergic diseases, such as in birch pollen- [170], house dust mite- [171] and cat dander- [163] mediated allergies. The T cell type supposed to become induced by peptide immunotherapy following persistent antigen stimulation is referred to as peptide-induced Treg. These piTregs are characterized by the phenotype CD25–CTLA-4+Foxp3− [172] and they reportedly express the transcription factors Tbet and Egr2 [173]. In principle, peptide immunotherapy represents an attractive therapeutic strategy, since it entirely focuses on T cell epitopes derived from major allergens (antigens), which are usually not recognized by the patients’ IgE [174] and therefore their application should not bear the risk of anaphylactic reactions triggered by type I immune reactions. Peptides derived from Fel d 1, the major cat dander allergen, have already been tested in clinical studies and, in fact, positively influenced the course of the disease [175–177].

Although, in principle, peptide immunotherapy (the same holds true for APL therapy) seems to be effective, the diverse set of HLA molecules in patients and thus the largely unpredictable course of peptide binding within the overall population of affected individuals represents a major caveat [169]. Consequently, the ensuing T cell responses to both wild-type and variant peptides still need to be empirically determined for each peptide immunogen designed for therapeutic application [178].

Moreover, peptide immunotherapy is also not completely devoid of adverse reactions, in fact, late-phase reactions causing airway hyperreactivity have been documented following the application of therapeutic peptides. In particular, in the first studies applying peptides from the cat allergen Fel d 1, patients developed postinjection symptoms upon treatment with Fel d 1 peptides [179] (table 3). This led to a change to the strategy for peptide design and to the use of shorter peptides in subsequent experiments [175, 176].

Apart from allergies, peptide (altered peptide) immunotherapy was also considered to be a possible therapeutic modality for otherwise hard-to-treat autoimmune diseases, such as MS [180]. Notably, Streeter et al. [181] developed a new peptide-based therapy for MS, consisting of a mixture of different T cell epitopes derived from MBP that ameliorated the disease when applied prophylactically and also therapeutically to Obx-DR2 mice.

Very similar to the binding and function of wild-type peptides derived from microbial sources, tumors or allergens, the functional effects induced by APLs also strongly depend on the immune response molecules, i.e. the HLA alleles, they are binding to and which affect their presentation to T cells. Consequently, while a given APL might function as a bona fide superagonist, partial agonist or even as an antagonist on a certain MHC background expressing the appropriate MHC allele, it might cause different, even opposite effects when introduced into another background.

While this is not surprising per se, it certainly demonstrates that APLs have to be regarded as precision medicine [182], which can be highly successful in genetically well-defined subgroups of individuals, but not necessarily in the overall population. In that respect the immunomodulatory behavior of APLs is not different from classical, immunodominant peptides. They also tend to unfold their full immunostimulatory repertoire only in the context of the appropriate restriction element(s). However, it requires that APLs become tailored to the individual patient(s), i.e. those expressing the appropriate but lacking possibly incompatible HLA alleles. Of note, some autoimmune and/or inflammatory diseases, such as RA or CD [78, 79, 183, 184], as well as a few allergies, such as allergy to mugwort pollen [132], show strong associations with small sets of HLA alleles.

With today’s high-resolution HLA-typing algorithms, this restriction does not seem to represent a major disadvantage of future APL therapies. In addition, efficacy might be further improved by the use of a mixture of APLs covering more than one important epitope within an immunodominant protein.

The identification of HLA alleles, e.g. in clinical trials, which might be incompatible with APL therapy, seems to be a more challenging task in that respect. Another possible disadvantage of APL-based therapies is their requirement for parenteral application by repeated injections. However, also this potential disadvantage could be dealt with, for instance with the appropriate formulation of peptides, e.g. by lipid coating (liposomes) increasing the peptides’ half-life in vivo [185].

Another unknown possibly influencing the functional effects of APLs is the molecular set-up of the APCs the APLs are binding to. APCs exist in different ‘flavors’ [186] and are well known to express and secrete different sets of activating and inhibitory molecules, which makes the prediction of functional outcomes induced by APLs upon binding to them a difficult task. In that respect, the APL dosing also comes into play since it will strongly influence the range and intensity of presentation by the different APCs present in the human body [181].

Clonal T cell populations are essential for the accurate molecular identification and characterization of APLs [2, 9]. However, it is also evident that the hierarchy of the TCR contact sites defined by such means needs to be meticulously evaluated with polyclonal T cell populations in further steps. In fact, previous research has shown that it cannot a priori be assumed that TCR contact site hierarchies defined by T cell clones are representative for polyclonal T cell populations [5, 187, 188]. On the contrary, such conclusions might give a distorted impression and instead prevent the selection of the APL most suitable for therapeutic approaches [189].

## Preclinical and Clinical Trials Using APLs

Soon after the first in vitro studies describing the hitherto unprecedented functional effects and thus potential therapeutic possibilities offered by APLs, MBP-specific APLs were taken to clinical trials in an effort to target the immune response in MS patients. In fact, preceding experiments in animal models had shown great promise in experimental autoimmune encephalomyelitis, a rodent disease mimicking to some degree MS in humans [8, 35]. A key feature of the murine and also the human disease is the T cell response to MBP along with the production of Th1 cytokines. As early as 1994, Karin et al. [8] showed that altering one peptide residue within MBP 87–99 (p.K91A), representing a critical site for TCR recognition, resulted in a clear-cut reduction of TNF-α and IFN-γ production, resulting in the amelioration of the disease. In 1995, Nicholson et al. [35] confirmed these initial findings with a different APL for MBP_139–151_ (p.W144Q), and also clearly demonstrated that the altered Th response and not a mere HLA blocking mechanism is responsible for the observed beneficial effects.

Therefore, antagonistic APLs were expected to block the pathognomonic Th1 T cell responses, while partial agonists were expected to function through ‘bystander suppression’ by inducing the development of regulatory T cells. However, in the year 2000 the results of two phase II clinical trials in which two different APLs for MBP_83–99_ were applied were reported [188, 190] and, unexpectedly, the results differed from expectations in the collectives of MS patients under study (table 3). The first of these trials applied the APL NBI-5788 (p.A91K) subcutaneously at 5, 20 or 50 mg/week for 9 months and included 144 patients. The trial had to be discontinued before it was concluded because approximately 10% of the patients developed systemic hypersensitivity-type reactions that included paresthesias in the extremities, exanthematous rash, dyspnea, nausea, abdominal pain and others (see table 3). Nevertheless, within the 53 patients that completed the double-blind phase of the trial, some improvement was registered in the group receiving the lower 5 mg/week dose of APL. As a matter of fact, the analysis by MRI revealed a reduction in the mean volume of brain lesions in this group, while the volume of the lesions was increased in the placebo-control group (a decrease of 207 ± 471 ml vs. an increase of 291 ± 698 ml). The immunological data were studied only in a small cohort of 7 patients but they showed a clear immune deviation towards a Th2 phenotype in the patients receiving the 5-mg/week treatment. However, the groups receiving higher doses neither showed improvement nor worsening of the disease, but they presented with most of the adverse effects as described above [190]. The second trial, which included only 8 patients and applied APL CGP77116, resulted in a complete failure. Instead of the generation of a protective Th2 response, a substantial expansion of Th1 cells with specificity for the APL that cross-reacted with MBP [188] was observed in all of the treated patients. In fact, in 2 of the treated patients the strong expansion of APL-specific T cells together with the weak expansion of MBP-specific T cells could be linked to the observed exacerbation of MS. All exacerbations could be treated by intravenous steroids. Nevertheless, these results cast great concern with regard to the overall design of the therapy.

Retrospectively, the surprising and unexpected failure of the trials was mainly attributed to the extremely high doses of APL administered to the patients in some groups, consisting, in fact, of weekly subcutaneous doses of 20 or 50 mg of APL. It is well known from the literature, that the engagement of a large number of TCRs due to high numbers of specific pMHCs present tends to promote Th1-biased immune responses [34, 191–193]. By the same token, the intentionally induced Th2 responses had apparently only a minor positive effect on the volume of enhancing MRI lesions [188, 190].

Moreover, the failures were also attributed to the fact that single APLs might not necessarily be able to antagonize all pathognomonic T cells with the same epitope specificity but deriving from different clonotypes. Despite these initial drawbacks, new attempts to exploit the beneficial effects of APLs have been made recently. For example, in a mouse encephalomyelitis model an APL derived from MBP_87–99_ was successfully applied to ameliorate chronic pain [194]. Notably, this APL also reduced mechanical pain hypersensitivity following peripheral nerve injury in Lewis rats when applied as a single dose of 250 μg [195].

Notwithstanding these dramatic initial failures of therapeutic trials applying APLs, a more recent phase I clinical trial conducted by Wraith and coworkers [181] using ATX-MS-1467, a mix of 5 different peptides derived from MBP, was successfully completed in 6 patients in which the doses applied were escalated from 25 to 800 μg either weekly or biweekly. The treatment was found to be safe and well tolerated by the patients enrolled, who were all suffering from secondary progressive MS. Of importance, no worsening of disease progression was observed in these patients. When compared to study entry, the immunological status of the different patients pointed to a reduction in the MBP-specific T cell responses [181].

In a related topic, Cortes et al. [196] reported the successful use of APLs in experimental autoimmune uveitis. Although the APLs were not intended to be used as a potential therapeutic peptide, they clearly provided proof of function. Priming of T cells with such peptides shifted them towards a Th2 phenotype and also induced Treg cells, which conferred protection from the disease.

## Conclusions

Dysregulated helper but also cytotoxic T cells might be the cause but also the consequence of autoimmune and allergic diseases. Hence, antigen-specific treatment modalities to counteract dysregulation and to turn off pathologic, lymphocyte-driven processes are highly desirable. The APL concept and related therapeutic principles of influencing/modifying T cell activation and polarization might thus pave the way for novel treatment modalities in the future. A ‘conditio sine qua non’ is certainly the availability of humanized test models (humanized mice) to test and monitor the effects of systemic application of such remedies.

## Figures and Tables

**Table 1:** Model peptides used to study APL function

Peptide	MHC	Role described or differential T cell function elicited by APLs	References
*Murine (in vitro)*			
Hb(64 – 76)	I-E^k^	IL-4 production and T cell-B cell collaboration (as assessed by B cell proliferation and Ig production) by Th2 clones; cytolytic activity, upregulation of CD25 and of LFA-1 and increased cell volume by Th1 clones; anergy in Th1 and Th2 clones	1, 10, 28, 37
PCC(88 – 104)	I-E^k^	Inhibition of IL-2 production by Th1 clones; upregulation of CD25 and increased cell volume; negative selection of DP thymocytes	12, 13
OVA(257 – 264)	K^b^	TCR antagonism of cytolysis, cytokine production, Ca^2+^ flux and serine esterase release in Th1 clones; induction of positive selection of thymocytes	4, 6, 48
LCMV GP(33 – 41)	H-2D^b^	Induction of positive selection of thymocytes and cytotoxic T cell memory	49, 197
MCC(88 – 103)	H-2E^k^	Inhibition of IL-2 production, upregulation of CD25 and increased cell volume; negative selection of DP thymocytes	13
huColIV	I-A^s^, I-A^b^	Differential IFN-γ and IL-4 production and T cell-B cell collaboration (as assessed by Ig production)	11

*Human (in vitro)*			
HA(307 – 319)	HLA-DR1	TCR antagonism causing reduced proliferation of Th1 clones	3
TT(830 – 843)	HLA-DR1	TCR antagonism causing reduced proliferation of Th1 clones	3
HA(307 – 319)	HLA-DR5	TCR antagonism as antigen-specific process	5
HBc(18 – 27)	HLA-A2	Naturally occurring TCR antagonists: antagonism of cytolytic function	64

PCC = Pigeon cytochrome C; LCMV-GP = lymphocytic choriomeningitis virus-Gag protein; DP = double positive; MCC = moth cytochrome C; HA = influenza hemagglutinin; TT = tetanus toxoid; HBc = hepatitis B virus core; huColIV = human collagen IV.

**Table 2:** APL of immunodominant allergen peptides

Allergen source	Peptide	HLA	Differential T cell response observed	Ref.
Japanese cedar *(Cryptomeria japonica)* pollen	Cry j 1_335 – 346_	DRB3*0301	p.T339G and p.T339Q induced 70% less T cell proliferation, and no IL-4, IL-2 or IFN-γ. p.T339V induced more IFN-γ than wild-type. 339Thr ‘hot-spot’ for altered T cell recognition in Cry j 1	148
House dust mite *(Dermatophagoides pteronyssinus)*	Der p 1_94 – 104_ Der p 1_171 – 182_	DRB1*1101 DRB1*1501	3/4 of Der p 1_94 – 104_ variants (p.R95A, p.Y96A and p.Q101A) and 2/6 of Der p 1_171 – 182_ variants (p.N173A and p.Q181A >50%) antagonized proliferation induced by wild-type peptides. p.R95A and p.Y96A as well as p.N173A and p.Q181A also blocked IL-2 and IFN-γ, but not IL-4 production. T cell clones stimulated with wild-type peptide in the presence of p.Y96A provide less help to B cells for IgE production, reflected by less CD40L expression	149
Spreading pellitory *(Parietaria judaica)* pollen	Par j 1_47 – 65_	DRB1*01 DRB1*03 DRB1*04	p.K52V, p.I57A and p.K60A represent poor stimulators of T cell proliferation, all bind with similar albeit lower affinity compared to wild-type to HLA molecules examined, but only p.K52V and p.I57A inhibit T cell proliferation	153
Bee venom	PLA_81 – 92_	DPB1	p.F82A, p.V83A, p.K85A, p.Y87A and p.L90A revealed significantly reduced proliferation and cytokine production. p.F82A led to an inverse IL-4/IFN-γ ratio due to preferential IL-4 inhibition. Preincubation with p.F82A induced anergy-specific T cells accompanied by reduced ZAP70 phosphorylation upon restimulation with mAb to CD3/CD28	154
Domestic cattle *(Bos domesticus)*	Bos d 2_127 – 142_	DRB1*0401	p.N135D and p.N133K stimulate T cell clones at lower concentrations than wild-type peptide (TCR modulation, CD25 neo-expression). Both APLs induce an increase in the IL-4/IFN-γ ratio. Both APLs induce increased cell death upon culture for 10 days with T cell clones	157, 158, 210

**Table 3:** Clinical trials: peptide and APL immunotherapy

Disease	Peptide	Patients, dosing and route	Proposed mechanism of action and outcome	References
*Peptide immunotherapy*				
Perennial allergic rhinitis and asthma	ALLERVAX CAT (Fel d 1_7 – 33_, Fel d 1_29 – 55_)	n = 95 each peptide at 7.5, 75 or 750 μg/week 4 SC doses	Tolerization of T cells by high concentrations of peptide in the absence of APC. Maintains IFN-γ, but stops IL-4 and IL-2 production. *Outcome:* reduced NSS and LSS compared to placebo (–2.3 ± 4.9 vs. –0.8 ± 0.5 and –2.3 ± 0.6 vs. –0.8 ± 0.6, respectively). Fel d 1-specific IgE and IgG unchanged after 6 weeks of treatment (750-μg dose). AE (750-μg group) included chest tightness, nasal congestion and itchy eyes (16 out of 24 patients)	179, 198

Perennial allergic rhinitis and asthma	FC1P (Fel d 1_11 – 27_, Fel d 1_22 – 37_, Fel d 1_28 – 44_)	n = 40 1 × 40 μg	ID immunization with T cell epitope-containing peptides induces late skin reactions independently of IgE thereby inducing tolerization to the allergen. *Outcome:* 9 patients displayed significantly decreased FEV_1_ after 2 – 6 h of treatment, without showing immediate or late skin reactions to the peptides. A second dose of 80 μg was applied after 2 – 6 weeks or 12 months in 6 patients (3 and 3, respectively). The first group showed reduced LARs while the second 3 displayed LARs of similar magnitude to those after the initial dose	199

Perennial allergic rhinitis and asthma	12 Fel d 1 peptides (16 – 17 mers) overlapping by 4 – 10 residues	n = 24 1, 2.5 or 5 μg 1 or 2 doses in 2 – 14 weeks ID	Tolerance induction preceded by strong T cell activation. Hyporesponsiveness upon secondary stimulation through intramolecular epitope, bystander or infectious suppression. *Outcome:* dose-dependent FEV_1_ reduction and loss of LAR with second dose in patients that presented with LAR after first dose. Reduction of IL-4, IL-13 and IFN-γ production by PBMC from the patients after 1 dose	200

Perennial allergic rhinitis and asthma	12 Fel d 1 peptides (16 – 17 mers) overlapping by 4 – 10 residues	n = 24 total dose: 0 μg^a^ ID	Same principle as above. *Outcome:* significant differences only between baseline and two follow-up treatment groups (4 – 8 weeks and 8 – 9 months) but not compared to placebo: increase of IL-10 and reduction of IL-4, IL-13 and IFN-γ production by PBMC; reduction of LAR to whole cat dander and Fel d 1 in both follow-ups; reduction of the early cutaneous reaction to Fel d 1 at second follow-up	201

Perennial allergic rhinitis and asthma	11 Fel d 1 peptides (16 – 17 mers) overlapping by 5 – 10 residues	n = 8 total dose: 41.1 μg^b^ ID	Tolerization with short overlapping peptides should induce a distinct Th cell recruitment to the allergen-challenge sites. *Outcome:* decreased airway hyperresponsiveness and inhibition of late-phase cutaneous reactions to whole cat allergen. Increase of cutaneous CD4+IFN-γ + and CD4+CD25+ cells but not of CD4+IL-10+ or CD4+CTLA-4+ cells at injection site (24 h)	202

Perennial allergic rhinitis and asthma	12 Fel d 1 peptides (16 – 17mer) overlapping by 4 – 10 residues	n = 28 total dose: ≥291 μg^c^ ID	*Outcome:* significant improvement of LAR (p = 0.03) but not EAR as assessed by FEV_1_ 3 – 4 months after treatment in the treatment group as compared to baseline values but not between the treatment and placebo groups. Improvement in the QOL asthma and rhinitis score in the treatment group as compared to baseline values	203

Perennial allergic rhinitis and conjunctivitis	Cat-PAD (Fel d 1_40 – 55_, Fel d 1_56 – 71_, Fel d 1_95 – 107_, Fel d 1_115 – 130_, Fel d 1_121 – 137_, Fel d 1_131 – 147_, Fel d 1_146 – 161_)	n = 202 peptide-mix 4 × 75 μg monthly or 8 × 37.5 μg biweekly ID	Suppression of IL-4, IL-13 and IFN-γ and enhancement of IL-10 production creates an environment beneficial for Treg polarization. *Outcome:* improvement in TRSS compared to placebo after 18 – 22 (–5.4 ± 5.8 vs. –2.8 ± 5.3) and 50 – 54 (–6.8 ± 5.7 vs. –2.9 ± 5.6) weeks of treatment. No AE. Follow-up: reduction in TRSS compared to placebo after 1 (–7.1 ± 7.7 vs. –3.0 ± 5.6) and 2 (–5.9 ± 9.5 vs. –2.0 ± 5.7) years as assessed in EEC and TNSS	175, 176

Perennial allergic rhinitis and asthma	Amb a 1 (3 peptides)	n = 960 75 or 750 μg 1 – 2 times/week for 2 weeks	Immunization with long peptides comprising several T cell epitopes, conferring possible protection on population level. *Outcome:* significant reduction of RCS and NSS compared to placebo, reduction of the mean TSS during the overall season. AE comprised immediate responses in <1% of the patients	204

Bee venom allergy	Api m 1_45 – 62_, Api m 1_82 – 92_, Api m 1_113 – 124_	n = 5 initial total dose: 97.1 μg^d^ maintenance dose: 100 μg/week for 3 weeks SC	Induction of tolerance to the whole allergen by inducing T cell anergy and by decreasing the IgE/IgG4-ratio. *Outcome:* suppression of IL-2, IL-4, IL-5, IL-13 and IFN-γ produced by PBMC after 2 months of treatment in 3 of 5 patients. Significant increase of IgE and IgG4 favoring IgG4 after 2 months of treatment. AE only in patients without improvement (mild angioedema lips and eyelids, erythema on face, urticarial wheels on chest and thighs)	205

Bee venom allergy	Api m 1_1 – 60_, Api m 1_47 – 99_, Api m 1_90 – 134_	n = 9 initial total dose: 251 μg^e^ maintenance dose: 5 × 100 (or 300) μg during next 70 days ID	Long overlapping peptides to overcome the high variation between epitope restriction. Avoidance of IgE response but induction of T cell hyporesponsiveness and immune deviation. *Outcome:* induction of T cell anergy and of IFN-γ- and IL-10- (but not Th2 cytokine) producing T cells after 42 days of treatment. Increase of specific IgG4 but not IgE levels. AE included pruritus (palms) and erythema (trunk)	206

Bee venom allergy	Api m 1_45 – 62_, Api m 1_81 – 92_, Api m 1_113 – 124_	n = 12 initial total dose: 131.1 μg^f^ maintenance dose: 3 × 100 μg (weekly) SC	High affinity peptides for collection of HLA molecules commonly expressed in the population. *Outcome:* induction of T cell anergy and IL-10 production, and reduction of IL-13 production after 12 weeks of treatment. Reduction of cutaneous late-phase reactions to allergen challenge. Transient and mild increase of allergen-specific IgG	207, 208

Secondary progressive multiple sclerosis	ATX-MS-1467 (MBP_30 – 44_, MBP_83 – 99_, MBP_130 – 144_, MBP_140 – 154_, MBP_156 – 170_)	n = 6 initial total dose: 575 μg^g^ maintenance dose: 2 × 800 μg weekly or biweekly ID	Repetitive exposure to the antigenic peptide induced hyporesponsiveness of the Th1 polarized T cells. *Outcome:* the treatment was well tolerated, there was no change in the EDSS or development of antibody directed against the peptides administered in any patient. Reduction of the T cell proliferation in response to MBP 1 month after the last dose. IL-10 mRNA increase dependent on HLA background. AE: muscle spasms and decreased ability in both legs (n = 2)	181

*APL immunotherapy*				
Multiple sclerosis	NBI-5788 (MBP_83 – 99_)	n = 14 5, 10 or 20 mg weekly for 4 weeks SC	*Outcome:* increased frequency of APL-responsive T cells compared to placebo (35.8 ± 12.8 vs. 6.2 ± 1.3%). APL-specific TCLs from treated patients were predominantly Th2 and showed increased cross-reactivity with MBP_93 – 99_ when compared to APL-specific TCL from untreated patients	209

Relapsingremitting multiple sclerosis	NBI-5788 (MBP_83 – 99_)	n = 144^h^ 5, 20 or 50 mg weekly for 16 weeks SC	APL supposed to induce a protective Th2 response. *Outcome:* existing MRI lesions reduced (5-mg dose: <207 ± 471 ml vs. placebo: >291 ± 698 ml), and patients with new MRI lesions reduced (57%, n = 14, vs. 25%, n = 16) after 4 months of treatment. Immune deviation (n = 7) and induction of IL-5 and IL-13 but not IFN-γ after 4 – 8 weeks of treatment (5-mg dose). AE in 10% of the patients led to suspension of the study. AE included among others itching, paresthesias (extremities), macules (trunk), exanthematous rash, dyspnea, nausea, abdominal pain, eosinophilia and hives. Anti-IgG titers against NBI-5788 increased in patients showing AE	190

Multiple sclerosis	CGP77116 (MBP_83 – 99_)	n = 8 50 mg weekly for 36 weeks^i^ SC	The APL may block the MS-specific T cell response by acting as a partial agonist, and/or antagonist or through bystander suppression. APL supposed to induce a protective Th2 response. *Outcome:* MRI-enhanced lesions unchanged in number. Numbers of APL-responsive T cells were clearly increased (2-fold) in all patients. APL-specific TCL revealed increased Th1/Th0 ratios due to increased IFN-γ (mean values: 636 vs. 495 pg/ml) and reduced IL-4 production (mean values: 69 vs. 158 pg/ml). The percentage of APL-specific TCL cross-reacting with MBP_83 – 99_ was increased from 6.9 to 37.6%. AE included 3 MS exacerbations and systemic hypersensitivity reactions to the APL	188

AE = Adverse effects; BSC = bee sting challenge; EAR = early asthma reaction; EEC = environmental exposure chamber; FEV_1_ = forced expiratory volume in 1 s; ID = intradermal; LAR = late asthma reaction; LSS = lung symptom score; MRI = magnetic resonance imaging; NSS = nasal symptom score; QOL = quality of life; RCS = rhinoconjunctival symptom score; SC = subcutaneous; TCL = T cell line; TRSS = total rhinoconjunctivitis symptom score; TSS = total symptom score.

a Four escalating doses (5, 10, 25 and 50 μg) applied at 3- to 4-day intervals.

b Five escalating doses (0.1, 1, 5, 10, 25 μg) applied at biweekly intervals.

c Seven escalating doses (1, 5, 10, 25, 50, 100, 100 μg) at biweekly intervals, when indicated with repetitions.

d Seven escalating doses (0.1 1, 3, 6, 12, 25, 50 μg) applied at 30-min intervals.

e Seven escalating doses (0.1, 1, 10, 20, 40, 80, 100 μg) of each of the three peptides applied at 30-min intervals.

f Six escalating doses (0.1, 1, 5, 25,50, 50 μg) of each of the 3 peptides applied weekly.

g Four escalating doses (25, 50, 100, 400 μg) of the peptide mixture in weekly or biweekly intervals.

h Only 53 patients completed the double-blind phase and the results correspond to those patients.

i From patient No. 8 onwards treatment was changed to 5 mg weekly for 1 month followed by 5 mg monthly for 8 months.

## References

[1] Evavold BD, Allen PM (1991). Separation of IL-4 production from Th cell proliferation by an altered T cell receptor ligand. Science.

[2] Evavold BD, Williams SG, Hsu BL, Buus S, Allen PM (1992). Complete dissection of the Hb(64–76) determinant using T helper 1, T helper 2 clones, and T cell hybridomas. J Immunol.

[3] De Magistris MT, Alexander J, Coggeshall M, Altman A, Gaeta FC, Grey HM, Sette A (1992). Antigen analog-major histocompatibility complexes act as antagonists of the T cell receptor. Cell.

[4] Jameson SC, Carbone FR, Bevan MJ (1993). Clone-specific T cell receptor antagonists of major histocompatibility complex class I-restricted cytotoxic T cells. J Exp Med.

[5] Ostrov D, Krieger J, Sidney J, Sette A, Concannon P (1993). T cell receptor antagonism mediated by interaction between T cell receptor junctional residues and peptide antigen analogues. J Immunol.

[6] Hogquist KA, Jameson SC, Heath WR, Howard JL, Bevan MJ, Carbone FR (1994). T cell receptor antagonist peptides induce positive selection. Cell.

[7] Wauben MH, Boog CJ, van der Zee R, Joosten I, Schlief A, van Eden W (1992). Disease inhibition by major histocompatibility complex binding peptide analogues of disease-associated epitopes: more than blocking alone. J Exp Med.

[8] Karin N, Mitchell DJ, Brocke S, Ling N, Steinman L (1994). Reversal of experimental autoimmune encephalomyelitis by a soluble peptide variant of a myelin basic protein epitope: T cell receptor antagonism and reduction of interferon γ and tumor necrosis factor α production. J Exp Med.

[9] Evavold BD, Sloan-Lancaster J, Allen PM (1993). Tickling the TCR: selective T-cell functions stimulated by altered peptide ligands. Immunol Today.

[10] Sloan-Lancaster J, Evavold BD, Allen PM (1993). Induction of T-cell anergy by altered T-cell-receptor ligand on live antigen-presenting cells. Nature.

[11] Pfeiffer C, Stein J, Southwood S, Ketelaar H, Sette A, Bottomly K (1995). Altered peptide ligands can control CD4 T lymphocyte differentiation in vivo. J Exp Med.

[12] Racioppi L, Ronchese F, Matis LA, Germain RN (1993). Peptide-major histocompatibility complex class II complexes with mixed agonist/antagonist properties provide evidence for ligand-related differences in T cell receptor-dependent intracellular signaling. J Exp Med.

[13] Page DM, Alexander J, Snoke K, Appella E, Sette A, Hedrick SM, Grey HM (1994). Negative selection of CD4+ CD8+ thymocytes by T-cell receptor peptide antagonists. Proc Natl Acad Sci USA.

[14] Robertson JM, Evavold BD (1999). Cutting edge: dueling TCRs: peptide antagonism of CD4+ T cells with dual antigen specificities. J Immunol.

[15] Dittel BN, Stefanova I, Germain RN, Janeway CA (1999). Cross-antagonism of a T cell clone expressing two distinct T cell receptors. Immunity.

[16] Yang W, Grey HM (2003). Study of the mechanism of TCR antagonism using dual-TCR-expressing T cells. J Immunol.

[17] Jones DS, Reichardt P, Ford ML, Edwards LJ, Evavold BD (2008). TCR antagonism by peptide requires high TCR expression. J Immunol.

[18] Stefanova I, Hemmer B, Vergelli M, Martin R, Biddison WE, Germain RN (2003). TCR ligand discrimination is enforced by competing ERK positive and SHP-1 negative feedback pathways. Nat Immunol.

[19] Wylie DC, Das J, Chakraborty AK (2007). Sensitivity of T cells to antigen and antagonism emerges from differential regulation of the same molecular signaling module. Proc Natl Acad Sci USA.

[20] Lafferty KJ, Cunningham AJ (1975). A new analysis of allogeneic interactions. Aust J Exp Biol Med Sci.

[21] Sloan-Lancaster J, Allen PM (1995). Significance of T-cell stimulation by altered peptide ligands in T cell biology. Curr Opin Immunol.

[22] Sloan-Lancaster J, Shaw AS, Rothbard JB, Allen PM (1994). Partial T cell signaling: altered phospho-ζ and lack of zap70 recruitment in APL-induced T cell anergy. Cell.

[23] Rabinowitz JD, Beeson C, Wulfing C, Tate K, Allen PM, Davis MM, McConnell HM (1996). Altered T cell receptor ligands trigger a subset of early T cell signals. Immunity.

[24] McConnell HM, Owicki JC, Parce JW, Miller DL, Baxter GT, Wada HG, Pitchford S (1992). The cytosensor microphysiometer: biological applications of silicon technology. Science.

[25] Boutin Y, Leitenberg D, Tao X, Bottomly K (1997). Distinct biochemical signals characterize agonist- and altered peptide ligand-induced differentiation of naive CD4+ T cells into Th1 and Th2 subsets. J Immunol.

[26] Kersh GJ, Kersh EN, Fremont DH, Allen PM (1998). High- and low-potency ligands with similar affinities for the TCR: the importance of kinetics in TCR signaling. Immunity.

[27] Sloan-Lancaster J, Allen PM (1996). Altered peptide ligand-induced partial T cell activation: molecular mechanisms and role in T cell biology. Annu Rev Immunol.

[28] Sloan-Lancaster J, Evavold BD, Allen PM (1994). Th2 cell clonal anergy as a consequence of partial activation. J Exp Med.

[29] Sloan-Lancaster J, Steinberg TH, Allen PM (1996). Selective activation of the calcium signaling pathway by altered peptide ligands. J Exp Med.

[30] Ding L, Shevach EM (1998). Differential effects of CD28 engagement and IL-12 on T cell activation by altered peptide ligands. J Immunol.

[31] van Bergen J, Koning F (1998). Altered peptide ligands and wild-type peptide induce indistinguishable responses of a human Th0 clone. Eur J Immunol.

[32] Faber-Elmann A, Grabovsky V, Dayan M, Sela M, Alon R, Mozes E (2000). Cytokine profile and T cell adhesiveness to endothelial selectins: in vivo induction by a myasthenogenic T cell epitope and immunomodulation by a dual altered peptide ligand. Int Immunol.

[33] Hollsberg P, Weber WE, Dangond F, Batra V, Sette A, Hafler DA (1995). Differential activation of proliferation and cytotoxicity in human T-cell lymphotropic virus type I Tax-specific CD8 T cells by an altered peptide ligand. Proc Natl Acad Sci USA.

[34] Tao X, Grant C, Constant S, Bottomly K (1997). Induction of IL-4-producing CD4+ T cells by antigenic peptides altered for TCR binding. J Immunol.

[35] Nicholson LB, Greer JM, Sobel RA, Lees MB, Kuchroo VK (1995). An altered peptide ligand mediates immune deviation and prevents autoimmune encephalomyelitis. Immunity.

[36] Kersh GJ, Allen PM (1996). Structural basis for T cell recognition of altered peptide ligands: a single T cell receptor can productively recognize a large continuum of related ligands. J Exp Med.

[37] Evavold BD, Sloan-Lancaster J, Hsu BL, Allen PM (1993). Separation of T helper 1 clone cytolysis from proliferation and lymphokine production using analog peptides. J Immunol.

[38] Sette A, Vitiello A, Reherman B, Fowler P, Nayersina R, Kast WM, Melief CJM, Oseroff C, Yuan L, Ruppert J, Sidney J (1994). The relationship between class-I binding affinity and immunogenicity of potential cytotoxic T-cell epitopes. J Immunol.

[39] Lipford GB, Bauer S, Wagner H, Heeg K (1995). In vivo CTL induction with point-substituted ovalbumin peptides: immunogenicity correlates with peptide-induced MHC class I stability. Vaccine.

[40] Chen W, Khilko S, Fecondo J, Margulies DH, McCluskey J (1994). Determinant selection of major histocompatibility complex class I-restricted antigenic peptides is explained by class I-peptide affinity and is strongly influenced by non-dominant anchor residues. J Exp Med.

[41] Webb AI, Dunstone MA, Chen W, Aguilar MI, Chen Q, Jackson H, Chang L, Kjer-Nielsen L, Beddoe T, McCluskey J, Rossjohn J (2004). Functional and structural characteristics of NY-ESO-1-related HLA A2-restricted epitopes and the design of a novel immunogenic analogue. J Biol Chem.

[42] Parkhurst MR, Salgaller ML, Southwood S, Robbins PF, Sette A, Rosenberg SA, Kawakami Y (1996). Improved induction of melanoma-reactive CTL with peptides from the melanoma antigen gp100 modified at HLA-A*0201-binding residues. J Immunol.

[43] Borbulevych OY, Baxter TK, Yu ZY, Restifo NP, Baker BM (2005). Increased immunogenicity of an anchor-modified tumor-associated antigen is due to the enhanced stability of the peptide/MHC complex: implications for vaccine design. J Immunol.

[44] Denkberg G, Klechevsky E, Reiter Y (2002). Modification of a tumor-derived peptide at an HLA-A2 anchor residue can alter the conformation of the MHC-peptide complex: probing with TCR-like recombinant antibodies. J Immunol.

[45] Chen JL, Dunbar PR, Gileadi U, Jager E, Gnjatic S, Nagata Y, Stockert E, Panicali DL, Chen YT, Knuth A, Old LJ (2000). Identification of NY-ESO-1 peptide analogues capable of improved stimulation of tumor-reactive CTL. J Immunol.

[46] van Stipdonk MJ, Badia-Martinez D, Sluijter M, Offringa R, van Hall T, Achour A (2009). Design of agonistic altered peptides for the robust induction of CTL directed towards H-2Db in complex with the melanoma-associated epitope gp100. Cancer Res.

[47] Chen S, Li Y, Depontieu FR, McMiller TL, English AM, Shabanowitz J, Kos F, Sidney J, Sette A, Rosenberg SA, Hunt DF (2013). Structure-based design of altered MHC class II-restricted peptide ligands with heterogeneous immunogenicity. J Immunol.

[48] Jameson SC, Hogquist KA, Bevan MJ (1994). Specificity and flexibility in thymic selection. Nature.

[49] Ashton-Rickardt PG, Bandeira A, Delaney JR, Van Kaer L, Pircher HP, Zinkernagel RM, Tonegawa S (1994). Evidence for a differential avidity model of T cell selection in the thymus. Cell.

[50] Hsu BL, Evavold BD, Allen PM (1995). Modulation of T cell development by an endogenous altered peptide ligand. J Exp Med.

[51] Berkley AM, Fink PJ (2014). Cutting edge: CD8+ recent thymic emigrants exhibit increased responses to low-affinity ligands and improved access to peripheral sites of inflammation. J Immunol.

[52] Barbera A, Lorenzo N, Garrido G, Mazola Y, Falcon V, Torres AM, Hernandez MI, Hernandez MV, Margry B, de Groot AM, van Roon J (2013). APL-1, an altered peptide ligand derived from human heat-shock protein 60, selectively induces apoptosis in activated CD4+ CD25+ T cells from peripheral blood of rheumatoid arthritis patients. Int Immunopharmacol.

[53] Lorenzo N, Cantera D, Barbera A, Alonso A, Chall E, Franco L, Ancizar J, Nunez Y, Altruda F, Silengo L, Padron G (2015). APL-2, an altered peptide ligand derived from heat-shock protein 60, induces interleukin-10 in peripheral blood mononuclear cell derived from juvenile idiopathic arthritis patients and downregulates the inflammatory response in collagen-induced arthritis model. Clin Exp Med.

[54] Faber-Elmann A, Grabovsky V, Dayan M, Sela M, Alon R, Mozes E (2001). An altered peptide ligand inhibits the activities of matrix metal-loproteinase-9 and phospholipase C, and inhibits T cell interactions with VCAM-1 induced in vivo by a myasthenogenic T cell epitope. FASEB J.

[55] Paas-Rozner M, Sela M, Mozes E (2001). The nature of the active suppression of responses associated with experimental autoimmune myasthenia gravis by a dual altered peptide ligand administered by different routes. Proc Natl Acad Sci USA.

[56] Paas-Rozner M, Sela M, Mozes E (2003). A dual altered peptide ligand down-regulates myasthenogenic T cell responses by up-regulating CD25- and CTLA-4-expressing CD4+ T cells. Proc Natl Acad Sci USA.

[57] Ben-David H, Sharabi A, Dayan M, Sela M, Mozes E (2007). The role of CD8+CD28 regulatory cells in suppressing myasthenia gravis-associated responses by a dual altered peptide ligand. Proc Natl Acad Sci USA.

[58] Ben-David H, Aruna BV, Seger R, Sela M, Mozes E (2006). A 50-kDa ERK-like protein is up-regulated by a dual altered peptide ligand that suppresses myasthenia gravis-associated responses. Proc Natl Acad Sci USA.

[59] Ben-David H, Sela M, Mozes E (2005). Down-regulation of myasthenogenic T cell responses by a dual altered peptide ligand via CD4+CD25+-regulated events leading to apoptosis. Proc Natl Acad Sci USA.

[60] Norris PJ, Stone JD, Anikeeva N, Heitman JW, Wilson IC, Hirschkorn DF, Clark MJ, Moffett HF, Cameron TO, Sykulev Y, Stern LJ (2006). Antagonism of HIV-specific CD4+ T cells by C-terminal truncation of a minimum epitope. Mol Immunol.

[61] Purbhoo MA, Sewell AK, Klenerman P, Goulder PJ, Hilyard KL, Bell JI, Jakobsen BK, Phillips RE (1998). Copresentation of natural HIV-1 agonist and antagonist ligands fails to induce the T cell receptor signaling cascade. Proc Natl Acad Sci USA.

[62] Burroughs NJ, Rand DA (1998). Dynamics of T-cell antagonism: enhanced viral diversity and survival. Proc Biol Sci.

[63] Frasca L, Del Porto P, Tuosto L, Marinari B, Scotta C, Carbonari M, Nicosia A, Piccolella E (1999). Hypervariable region 1 variants act as TCR antagonists for hepatitis C virus-specific CD4+ T cells. J Immunol.

[64] Bertoletti A, Sette A, Chisari FV, Penna A, Levrero M, De Carli M, Fiaccadori F, Ferrari C (1994). Natural variants of cytotoxic epitopes are T-cell receptor antagonists for antiviral cytotoxic T cells. Nature.

[65] Gilbert SC, Plebanski M, Gupta S, Morris J, Cox M, Aidoo M, Kwiatkowski D, Greenwood BM, Whittle HC, Hill AV (1998). Association of malaria parasite population structure, HLA, and immunological antagonism. Science.

[66] McAdam SN, Fleckenstein B, Rasmussen IB, Schmid DG, Sandlie I, Bogen B, Viner NJ, Sollid LM (2001). T cell recognition of the dominant I-A(k)-restricted hen egg lysozyme epitope: critical role for asparagine deamidation. J Exp Med.

[67] Mamula MJ, Gee RJ, Elliott JI, Sette A, Southwood S, Jones PJ, Blier PR (1999). Isoaspartyl post-translational modification triggers autoimmune responses to self-proteins. J Biol Chem.

[68] Hill JA, Southwood S, Sette A, Jevnikar AM, Bell DA, Cairns E (2003). Cutting edge: the conversion of arginine to citrulline allows for a high-affinity peptide interaction with the rheumatoid arthritis-associated HLA-DRB1*0401 MHC class II molecule. J Immunol.

[69] Makrygiannakis D, af Klint E, Lundberg IE, Lofberg R, Ulfgren AK, Klareskog L, Catrina AI (2006). Citrullination is an inflammation-dependent process. Ann Rheum Dis.

[70] Kim JK, Mastronardi FG, Wood DD, Lubman DM, Zand R, Moscarello MA (2003). Multiple sclerosis: an important role for post-translational modifications of myelin basic protein in pathogenesis. Mol Cell Proteomics.

[71] Ireland J, Herzog J, Unanue ER (2006). Cutting edge: Unique T cells that recognize citrullinated peptides are a feature of protein immunization. J Immunol.

[72] Nakashima K, Hagiwara T, Ishigami A, Nagata S, Asaga H, Kuramoto M, Senshu T, Yamada M (1999). Molecular characterization of peptidylarginine deiminase in HL-60 cells induced by retinoic acid and 1α,25-dihydroxyvitamin D_3_. J Biol Chem.

[73] Ireland JM, Unanue ER (2011). Autophagy in antigen-presenting cells results in presentation of citrullinated peptides to CD4 T cells. J Exp Med.

[74] Tighe MR, Hall MA, Barbado M, Cardi E, Welsh KI, Ciclitira PJ (1992). HLA class II alleles associated with celiac disease susceptibility in a southern European population. Tissue Antigens.

[75] Sollid LM, Markussen G, Ek J, Gjerde H, Vartdal F, Thorsby E (1989). Evidence for a primary association of celiac disease to a particular HLA-DQ α/β heterodimer. J Exp Med.

[76] Schuppan D, Junker Y, Barisani D (2009). Celiac disease: from pathogenesis to novel therapies. Gastroenterology.

[77] Lundin KE, Scott H, Fausa O, Thorsby E, Sollid LM (1994). T cells from the small intestinal mucosa of a DR4, DQ7/DR4, DQ8 celiac disease patient preferentially recognize gliadin when presented by DQ8. Hum Immunol.

[78] Dieterich W, Ehnis T, Bauer M, Donner P, Volta U, Riecken EO, Schuppan D (1997). Identification of tissue transglutaminase as the autoantigen of celiac disease. Nat Med.

[79] Elli L, Bergamini CM, Bardella MT, Schuppan D (2009). Transglutaminases in inflammation and fibrosis of the gastrointestinal tract and the liver. Dig Liver Dis.

[80] Greenberg CS, Birckbichler PJ, Rice RH (1991). Transglutaminases: multifunctional crosslinking enzymes that stabilize tissues. FASEB J.

[81] Bruce SE, Bjarnason I, Peters TJ (1985). Human jejunal transglutaminase: demonstration of activity, enzyme kinetics and substrate specificity with special relation to gliadin and coeliac disease. Clin Sci (Lond).

[82] Molberg O, Kett K, Scott H, Thorsby E, Sollid LM, Lundin KE (1997). Gliadin specific, HLA DQ2-restricted T cells are commonly found in small intestinal biopsies from coeliac disease patients, but not from controls. Scand J Immunol.

[83] Lundin KE, Scott H, Hansen T, Paulsen G, Halstensen TS, Fausa O, Thorsby E, Sollid LM (1993). Gliadin-specific, HLA-DQ(α1*0501,β1*0201) restricted T cells isolated from the small intestinal mucosa of celiac disease patients. J Exp Med.

[84] van de Wal Y, Kooy Y, van Veelen P, Pena S, Mearin L, Papadopoulos G, Koning F (1998). Selective deamidation by tissue transglutaminase strongly enhances gliadin-specific T cell reactivity. J Immunol.

[85] Molberg O, McAdam SN, Korner R, Quarsten H, Kristiansen C, Madsen L, Fugger L, Scott H, Noren O, Roepstorff P, Lundin KE (1998). Tissue transglutaminase selectively modifies gliadin peptides that are recognized by gut-derived T cells in celiac disease. Nat Med.

[86] Arentz-Hansen H, McAdam SN, Molberg O, Fleckenstein B, Lundin KE, Jorgensen TJ, Jung G, Roepstorff P, Sollid LM (2002). Celiac lesion T cells recognize epitopes that cluster in regions of gliadins rich in proline residues. Gastroenterology.

[87] Shan L, Molberg O, Parrot I, Hausch F, Filiz F, Gray GM, Sollid LM, Khosla C (2002). Structural basis for gluten intolerance in celiac sprue. Science.

[88] Vader W, Kooy Y, Van Veelen P, De Ru A, Harris D, Benckhuijsen W, Pena S, Mearin L, Drijfhout JW, Koning F (2002). The gluten response in children with celiac disease is directed toward multiple gliadin and glutenin peptides. Gastroenterology.

[89] Kim CY, Quarsten H, Bergseng E, Khosla C, Sollid LM (2004). Structural basis for HLA-DQ2-mediated presentation of gluten epitopes in celiac disease. Proc Natl Acad Sci USA.

[90] Henderson KN, Tye-Din JA, Reid HH, Chen Z, Borg NA, Beissbarth T, Tatham A, Mannering SI, Purcell AW, Dudek NL, van Heel DA (2007). A structural and immunological basis for the role of human leukocyte antigen DQ8 in celiac disease. Immunity.

[91] Fleckenstein B, Molberg O, Qiao SW, Schmid DG, von der Mulbe F, Elgstoen K, Jung G, Sollid LM (2002). Gliadin T cell epitope selection by tissue transglutaminase in celiac disease: role of enzyme specificity and pH influence on the transamidation versus deamidation process. J Biol Chem.

[92] Anderson RP, Degano P, Godkin AJ, Jewell DP, Hill AV (2000). In vivo antigen challenge in celiac disease identifies a single transglutaminase-modified peptide as the dominant A-gliadin T-cell epitope. Nat Med.

[93] Arentz-Hansen H, Korner R, Molberg O, Quarsten H, Vader W, Kooy YM, Lundin KE, Koning F, Roepstorff P, Sollid LM, McAdam SN (2000). The intestinal T cell response to α-gliadin in adult celiac disease is focused on a single deamidated glutamine targeted by tissue transglutaminase. J Exp Med.

[94] Qiao SW, Bergseng E, Molberg O, Xia J, Fleckenstein B, Khosla C, Sollid LM (2004). Antigen presentation to celiac lesion-derived T cells of a 33-mer gliadin peptide naturally formed by gastrointestinal digestion. J Immunol.

[95] Xia J, Sollid LM, Khosla C (2005). Equilibrium and kinetic analysis of the unusual binding behavior of a highly immunogenic gluten peptide to HLA-DQ2. Biochemistry.

[96] Xia J, Siegel M, Bergseng E, Sollid LM, Khosla C (2006). Inhibition of HLA-DQ2-mediated antigen presentation by analogues of a high affinity 33-residue peptide from α2-gliadin. J Am Chem Soc.

[97] Zarling AL, Ficarro SB, White FM, Shabanowitz J, Hunt DF, Engelhard VH (2000). Phosphorylated peptides are naturally processed and presented by major histocompatibility complex class I molecules in vivo. J Exp Med.

[98] Birnboim HC, Lemay AM, Lam DK, Goldstein R, Webb JR (2003). Cutting edge: MHC class II-restricted peptides containing the inflammation-associated marker 3-nitrotyrosine evade central tolerance and elicit a robust cell-mediated immune response. J Immunol.

[99] Stockl J, Majdic O, Fischer G, Maurer D, Knapp W (2001). Monomorphic molecules function as additional recognition structures on haptenated target cells for HLA-A1-restricted, hapten-specific CTL. J Immunol.

[100] Herzog J, Maekawa Y, Cirrito TP, Illian BS, Unanue ER (2005). Activated antigen-presenting cells select and present chemically modified peptides recognized by unique CD4 T cells. Proc Natl Acad Sci USA.

[101] Martin SF (2004). T lymphocyte-mediated immune responses to chemical haptens and metal ions: implications for allergic and autoimmune disease. Int Arch Allergy Immunol.

[102] Martin SF, Esser PR, Weber FC, Jakob T, Freudenberg MA, Schmidt M, Goebeler M (2011). Mechanisms of chemical-induced innate immunity in allergic contact dermatitis. Allergy.

[103] Pichler WJ, Adam J, Watkins S, Wuillemin N, Yun J, Yerly D (2015). Drug hypersensitivity: how drugs stimulate T cells via pharmacological interaction with immune receptors. Int Arch Allergy Immunol.

[104] Preckel T, Hellwig S, Pflugfelder U, Lappin MB, Weltzien HU (2001). Clonal anergy induced in a CD8+ hapten-specific cytotoxic T-cell clone by an altered hapten-peptide ligand. Immunology.

[105] Preckel T, Breloer M, Kohler H, von Bonin A, Weltzien HU (1998). Partial agonism and independent modulation of T cell receptor and CD8 in hapten-specific cytotoxic T cells. Eur J Immunol.

[106] Viner NJ, Nelson CA, Deck B, Unanue ER (1996). Complexes generated by the binding of free peptides to class II MHC molecules are antigenically diverse compared with those generated by intracellular processing. J Immunol.

[107] Viner NJ, Nelson CA, Unanue ER (1995). Identification of a major I-Ek-restricted determinant of hen egg lysozyme: limitations of lymph node proliferation studies in defining immunodominance and crypticity. Proc Natl Acad Sci USA.

[108] Peterson DA, DiPaolo RJ, Kanagawa O, Unanue ER (2001). Cutting edge: a single MHC anchor residue alters the conformation of a peptide-MHC complex inducing T cells that survive negative selection. J Immunol.

[109] Peterson DA, DiPaolo RJ, Kanagawa O, Unanue ER (1999). Quantitative analysis of the T cell repertoire that escapes negative selection. Immunity.

[110] Suri A, Lovitch SB, Unanue ER (2006). The wide diversity and complexity of peptides bound to class II MHC molecules. Curr Opin Immunol.

[111] Germain RN, Hendrix LR (1991). MHC class II structure, occupancy and surface expression determined by post-endoplasmic reticulum antigen binding. Nature.

[112] Neefjes JJ, Ploegh HL (1992). Inhibition of endosomal proteolytic activity by leupeptin blocks surface expression of MHC class II molecules and their conversion to SDS resistance alpha beta heterodimers in endosomes. EMBO J.

[113] Lindner R, Unanue ER (1996). Distinct antigen MHC class II complexes generated by separate processing pathways. EMBO J.

[114] Cirrito TP, Pu Z, Deck MB, Unanue ER (2001). Deamidation of asparagine in a major histocompatibility complex-bound peptide affects T cell recognition but does not explain type B reactivity. J Exp Med.

[115] Sanderson F, Kleijmeer MJ, Kelly A, Verwoerd D, Tulp A, Neefjes JJ, Geuze HJ, Trowsdale J (1994). Accumulation of HLA-DM, a regulator of antigen presentation, in MHC class II compartments. Science.

[116] Peters PJ, Neefjes JJ, Oorschot V, Ploegh HL, Geuze HJ (1991). Segregation of MHC class II molecules from MHC class I molecules in the Golgi complex for transport to lysosomal compartments. Nature.

[117] Harding CV (1995). Intracellular organelles involved in antigen processing and the binding of peptides to class II MHC molecules. Semin Immunol.

[118] Falk K, Lau JM, Santambrogio L, Esteban VM, Puentes F, Rotzschke O, Strominger JL (2002). Ligand exchange of major histocompatibility complex class II proteins is triggered by H-bond donor groups of small molecules. J Biol Chem.

[119] Gupta S, Hopner S, Rupp B, Gunther S, Dickhaut K, Agarwal N, Cardoso MC, Kuhne R, Wiesmuller KH, Jung G, Falk K (2008). Anchor side chains of short peptide fragments trigger ligand-exchange of class II MHC molecules. PLoS One.

[120] Afridi S, Shaheen F, Roetzschke O, Shah ZA, Abbas SC, Siraj R, Makhmoor T (2015). A cyclic peptide accelerates the loading of peptide antigens in major histocompatibility complex class II molecules. Biochem Biophys Res Commun.

[121] Hopner S, Dickhaut K, Hofstatter M, Kramer H, Ruckerl D, Soderhall JA, Gupta S, Marin-Esteban V, Kuhne R, Freund C, Jung G (2006). Small organic compounds enhance antigen loading of class II major histocompatibility complex proteins by targeting the polymorphic P1 pocket. J Biol Chem.

[122] Call MJ, Xing X, Cuny GD, Seth NP, Altmann DM, Fugger L, Krogsgaard M, Stein RL, Wucherpfennig KW (2009). In vivo enhancement of peptide display by MHC class II molecules with small molecule catalysts of peptide exchange. J Immunol.

[123] Govorkova EA, Fang HB, Tan M, Webster RG (2004). Neuraminidase inhibitor-rimantadine combinations exert additive and synergistic anti-influenza virus effects in MDCK cells. Antimicrob Agents Chemother.

[124] Jing X, Ma C, Ohigashi Y, Oliveira FA, Jardetzky TS, Pinto LH, Lamb RA (2008). Functional studies indicate amantadine binds to the pore of the influenza A virus M2 proton-selective ion channel. Proc Natl Acad Sci USA.

[125] Augeri DJ, Robl JA, Betebenner DA, Magnin DR, Khanna A, Robertson JG, Wang A, Simpkins LM, Taunk P, Huang Q, Han SP (2005). Discovery and preclinical profile of Saxagliptin (BMS-477118): a highly potent, long-acting, orally active dipeptidyl peptidase IV inhibitor for the treatment of type 2 diabetes. J Med Chem.

[126] Villhauer EB, Brinkman JA, Naderi GB, Burkey BF, Dunning BE, Prasad K, Mangold BL, Russell ME, Hughes TE (2003). 1-[[(3-hydroxy-1-adamantyl)amino]acetyl]-2-cyano-(S)-pyrrolidine: a potent, selective, and orally bioavailable dipeptidyl peptidase IV inhibitor with antihyperglycemic properties. J Med Chem.

[127] Tanaka T, Camerini D, Seed B, Torimoto Y, Dang NH, Kameoka J, Dahlberg HN, Schlossman SF, Morimoto C (1992). Cloning and functional expression of the T cell activation antigen CD26. J Immunol.

[128] Sakurada C, Sakurada S, Hayashi T, Katsuyama S, Tan-No K, Sakurada T (2003). Degradation of endomorphin-2 at the supraspinal level in mice is initiated by dipeptidyl peptidase IV: an in vitro and in vivo study. Biochem Pharmacol.

[129] Morimoto C, Torimoto Y, Levinson G, Rudd CE, Schrieber M, Dang NH, Letvin NL, Schlossman SF (1989). 1F7, a novel cell surface molecule, involved in helper function of CD4 cells. J Immunol.

[130] Dickhaut K, Hoepner S, Eckhard J, Wiesmueller KH, Schindler L, Jung G, Falk K, Roetzschke O (2009). Enhancement of tumourspecific immune responses in vivo by ‘MHC loading-enhancer’ (MLE). PLoS One.

[131] Roy K, Naskar K, Ghosh M, Roy S (2014). Class II MHC/peptide interaction in *Leishmania donovani* infection: implications in vaccine design. J Immunol.

[132] Jahn-Schmid B, Radakovics A, Luttkopf D, Scheurer S, Vieths S, Ebner C, Bohle B (2005). Bet v 1142–156 is the dominant T-cell epitope of the major birch pollen allergen and important for cross-reactivity with Bet v 1-related food allergens. J Allergy Clin Immunol.

[133] Wangorsch A, Larsson H, Messmer M, Garcia-Moral A, Lauer I, Wolfheimer S, Schulke S, Bartra J, Vieths S, Lidholm J, Scheurer S (2016). Molecular cloning of plane pollen allergen Pla a 3 and its utility as diagnostic marker for peach associated plane pollen allergy. Clin Exp Allergy.

[134] Archila LD, Chow IT, McGinty JW, Renand A, Jeong D, Robinson D, Farrington ML, Kwok WW (2016). Ana o 1 and Ana o 2 cashew allergens share cross-reactive CD4^+^ T-cell epitopes with other tree nuts. Clin Exp Allergy.

[135] Kazatsky AM, Wood RA (2016). Classification of food allergens and cross-reactivity. Curr Allergy Asthma Rep.

[136] Kobayashi Y, Akiyama H, Huge J, Kubota H, Chikazawa S, Satoh T, Miyake T, Uhara H, Okuyama R, Nakagawara R, Aihara M, Hamada-Sato N (2016). Fish collagen is an important panallergen in the Japanese population. Allergy.

[137] Lukschal A, Wallmann J, Bublin M, Hofstetter G, Mothes-Luksch N, Breiteneder H, Pali-Scholl I, Jensen-Jarolim E (2016). Mimotopes for Api g 5, a relevant cross-reactive allergen, in the celery-mugwort-birch-spice syndrome. Allergy Asthma Immunol Res.

[138] Miyaji K, Okamoto N, Saito A, Yasueda H, Takase Y, Shimakura H, Saito S, Sakaguchi M (2016). Cross-reactivity between major IgE core epitopes on Cry j 2 allergen of Japanese cedar pollen and relevant sequences on Cha o 2 allergen of Japanese cypress pollen. Allergol Int.

[139] Westernberg L, Schulten V, Greenbaum JA, Natali S, Tripple V, McKinney DM, Frazier A, Hofer H, Wallner M, Sallusto F, Sette A (2016). T-cell epitope conservation across allergen species is a major determinant of immunogenicity. J Allergy Clin Immunol.

[140] Wuthrich B (1990). Birch pollen allergy in relation with hypersensitivity for various kinds of fruit. Hautarzt.

[141] Wuthrich B, Stager J, Johansson SG (1990). Celery allergy associated with birch and mugwort pollinosis. Allergy.

[142] Kleine-Tebbe J, Vogel L, Crowell DN, Haustein UF, Vieths S (2002). Severe oral allergy syndrome and anaphylactic reactions caused by a Bet v 1- related PR-10 protein in soybean, SAM22. J Allergy Clin Immunol.

[143] Ebner C, Hirschwehr R, Bauer L, Breiteneder H, Valenta R, Ebner H, Kraft D, Scheiner O (1995). Identification of allergens in fruits and vegetables: IgE cross-reactivities with the important birch pollen allergens Bet v 1 and Bet v 2 (birch profilin). J Allergy Clin Immunol.

[144] Fritsch R, Bohle B, Vollmann U, Wiedermann U, Jahn-Schmid B, Krebitz M, Breiteneder H, Kraft D, Ebner C (1998). Bet v 1, the major birch pollen allergen, and Mal d 1, the major apple allergen, cross-react at the level of allergen-specific T helper cells. J Allergy Clin Immunol.

[145] Bohle B, Radakovics A, Jahn-Schmid B, Hoffmann-Sommergruber K, Fischer GF, Ebner C (2003). Bet v 1, the major birch pollen allergen, initiates sensitization to Api g 1, the major allergen in celery: evidence at the T cell level. Eur J Immunol.

[146] Bohle B, Radakovics A, Luttkopf D, Jahn-Schmid B, Vieths S, Ebner C (2005). Characterization of the T cell response to the major hazelnut allergen, Cor a 1.04: evidence for a relevant T cell epitope not cross-reactive with homologous pollen allergens. Clin Exp Allergy.

[147] Schimek EM, Zwolfer B, Briza P, Jahn-Schmid B, Vogel L, Vieths S, Ebner C, Bohle B (2005). Gastrointestinal digestion of Bet v 1-homologous food allergens destroys their mediator-releasing, but not T cell-activating, capacity. J Allergy Clin Immunol.

[148] Ikagawa S, Matsushita S, Chen YZ, Ishikawa T, Nishimura Y (1996). Single amino acid substitutions on a Japanese cedar pollen allergen (Cry j 1)-derived peptide induced alterations in human T cell responses and T cell receptor antagonism. J Allergy Clin Immunol.

[149] Fasler S, Aversa G, de Vries JE, Yssel H (1998). Antagonistic peptides specifically inhibit proliferation, cytokine production, CD40L expression, and help for IgE synthesis by Der p 1-specific human T-cell clones. J Allergy Clin Immunol.

[150] Weghofer M, Dall’Antonia Y, Grote M, Stocklinger A, Kneidinger M, Balic N, Krauth MT, Fernandez-Caldas E, Thomas WR, van Hage M, Vieths S (2008). Characterization of Der p 21, a new important allergen derived from the gut of house dust mites. Allergy.

[151] Weghofer M, Grote M, Resch Y, Casset A, Kneidinger M, Kopec J, Thomas WR, Fernandez-Caldas E, Kabesch M, Ferrara R, Mari A (2013). Identification of Der p 23, a peritrophin-like protein, as a new major *Dermatophagoides pteronyssinus* allergen associated with the peritrophic matrix of mite fecal pellets. J Immunol.

[152] Weghofer M, Thomas WR, Kronqvist M, Mari A, Purohit A, Pauli G, Horak F, Gronlund H, van Hage M, Valenta R, Vrtala S (2008). Variability of IgE reactivity profiles among European mite allergic patients. Eur J Clin Invest.

[153] De Palma R, Wu S, Sallusto F, Di Felice G, Martucci P, Geraci D, Colombo P, Troise C, Sacerdoti G, Nocera A, Gorski J (1999). Use of antagonist peptides to inhibit in vitro T cell responses to Par j1, the major allergen of *Parietaria judaica* pollen. J Immunol.

[154] Faith A, Akdis CA, Akdis M, Joss A, Wymann D, Blaser K (1999). An altered peptide ligand specifically inhibits Th2 cytokine synthesis by abrogating TCR signaling. J Immunol.

[155] Akdis CA, Blesken T, Akdis M, Wuthrich B, Blaser K (1998). Role of interleukin 10 in specific immunotherapy. J Clin Invest.

[156] DeSilva DR, Urdahl KB, Jenkins MK (1991). Clonal anergy is induced in vitro by T cell receptor occupancy in the absence of proliferation. J Immunol.

[157] Kinnunen T, Kwok WW, Narvanen A, Rytkonen-Nissinen M, Immonen A, Saarelainen S, Taivainen A, Virtanen T (2005). Immunomodulatory potential of heteroclitic analogs of the dominant T-cell epitope of lipocalin allergen Bos d 2 on specific T cells. Int Immunol.

[158] Kinnunen T, Buhot C, Narvanen A, Rytkonen-Nissinen M, Saarelainen S, Pouvelle-Moratille S, Rautiainen J, Taivainen A, Maillere B, Mantyjarvi R, Virtanen T (2003). The immunodominant epitope of lipocalin allergen Bos d 2 is suboptimal for human T cells. Eur J Immunol.

[159] Janssen EM, van Oosterhout AJ, van Rensen AJ, van Eden W, Nijkamp FP, Wauben MH (2000). Modulation of Th2 responses by peptide analogues in a murine model of allergic asthma: amelioration or deterioration of the disease process depends on the Th1 or Th2 skewing characteristics of the therapeutic peptide. J Immunol.

[160] Moldaver D, Larche M (2011). Immunotherapy with peptides. Allergy.

[161] Campbell JD, Buckland KF, McMillan SJ, Kearley J, Oldfield WL, Stern LJ, Gronlund H, van Hage M, Reynolds CJ, Boyton RJ, Cobbold SP (2009). Peptide immunotherapy in allergic asthma generates IL-10-dependent immunological tolerance associated with linked epitope suppression. J Exp Med.

[162] Moldaver DM, Bharhani MS, Wattie JN, Ellis R, Neighbour H, Lloyd CM, Inman MD, Larche M (2014). Amelioration of ovalbumin-induced allergic airway disease following Der p 1 peptide immunotherapy is not associated with induction of IL-35. Mucosal Immunol.

[163] Briner TJ, Kuo MC, Keating KM, Rogers BL, Greenstein JL (1993). Peripheral T-cell tolerance induced in naive and primed mice by subcutaneous injection of peptides from the major cat allergen Fel d I. Proc Natl Acad Sci USA.

[164] Critchfield JM, Racke MK, Zuniga-Pflucker JC, Cannella B, Raine CS, Goverman J, Lenardo MJ (1994). T cell deletion in high antigen dose therapy of autoimmune encephalomyelitis. Science.

[165] Critchfield JM, Zuniga-Pflucker JC, Lenardo MJ (1995). Parameters controlling the programmed death of mature mouse T lymphocytes in high-dose suppression. Cell Immunol.

[166] Gardner LM, O’Hehir RE, Rolland JM (2004). High dose allergen stimulation of T cells from house dust mite-allergic subjects induces expansion of IFN-γ+ T cells, apoptosis of CD4+IL-4+ T cells and T cell anergy. Int Arch Allergy Immunol.

[167] Liblau RS, Tisch R, Shokat K, Yang X, Dumont N, Goodnow CC, McDevitt HO (1996). Intravenous injection of soluble antigen induces thymic and peripheral T-cells apoptosis. Proc Natl Acad Sci USA.

[168] Suzuki G, Kawase Y, Koyasu S, Yahara I, Kobayashi Y, Schwartz RH (1988). Antigen-induced suppression of the proliferative response of T cell clones. J Immunol.

[169] Larche M, Wraith DC (2005). Peptide-based therapeutic vaccines for allergic and autoimmune diseases. Nat Med.

[170] Bauer L, Bohle B, Jahn-Schmid B, Wiedermann U, Daser A, Renz H, Kraft D, Ebner C (1997). Modulation of the allergic immune response in BALB/c mice by subcutaneous injection of high doses of the dominant T cell epitope from the major birch pollen allergen Bet v 1. Clin Exp Immunol.

[171] Hoyne GF, O’Hehir RE, Wraith DC, Thomas WR, Lamb JR (1993). Inhibition of T cell and antibody responses to house dust mite allergen by inhalation of the dominant T cell epitope in naive and sensitized mice. J Exp Med.

[172] Vieira PL, Christensen JR, Minaee S, O’Neill EJ, Barrat FJ, Boonstra A, Barthlott T, Stockinger B, Wraith DC, O’Garra A (2004). IL-10-secreting regulatory T cells do not express Foxp3 but have comparable regulatory function to naturally occurring CD4+CD25+ regulatory T cells. J Immunol.

[173] Anderson PO, Manzo BA, Sundstedt A, Minaee S, Symonds A, Khalid S, Rodriguez-Cabezas ME, Nicolson K, Li S, Wraith DC, Wang P (2006). Persistent antigenic stimulation alters the transcription program in T cells, resulting in antigen-specific tolerance. Eur J Immunol.

[174] Pellaton C, Perrin Y, Boudousquie C, Barbier N, Wassenberg J, Corradin G, Thierry AC, Audran R, Reymond C, Spertini F (2013). Novel birch pollen specific immunotherapy formulation based on contiguous overlapping peptides. Clin Transl Allergy.

[175] Couroux P, Patel D, Armstrong K, Larche M, Hafner RP (2015). Fel d 1-derived synthetic peptide immuno-regulatory epitopes show a long-term treatment effect in cat allergic subjects. Clin Exp Allergy.

[176] Patel D, Couroux P, Hickey P, Salapatek AM, Laidler P, Larche M, Hafner RP (2013). Fel d 1-derived peptide antigen desensitization shows a persistent treatment effect 1 year after the start of dosing: a randomized, place-bo-controlled study. J Allergy Clin Immunol.

[177] Verhoef A, Alexander C, Kay AB, Larche M (2005). T cell epitope immunotherapy induces a CD4+ T cell population with regulatory activity. PLoS Med.

[178] Leggatt GR (2014). Peptide dose and/or structure in vaccines as a determinant of T cell responses. Vaccines (Basel).

[179] Norman PS, Ohman JL, Long AA, Creticos PS, Gefter MA, Shaked Z, Wood RA, Eggleston PA, Hafner KB, Rao P, Lichtenstein LM (1996). Treatment of cat allergy with T-cell reactive peptides. Am J Respir Crit Care Med.

[180] Badawi AH, Siahaan TJ (2012). Immune modulating peptides for the treatment and suppression of multiple sclerosis. Clin Immunol.

[181] Streeter HB, Rigden R, Martin KF, Scolding NJ, Wraith DC (2015). Preclinical development and first-in-human study of ATX-MS-1467 for immunotherapy of MS. Neurol Neuroimmunol Neuroinflamm.

[182] Hamburg MA, Collins FS (2010). The path to personalized medicine. N Engl J Med.

[183] Gran JT, Husby G, Thorsby E (1983). The association between rheumatoid arthritis and the HLA antigen DR4. Ann Rheum Dis.

[184] Gran JT, Husby G, Thorsby E (1983). HLA DR antigens in rheumatoid arthritis. Scand J Rheumatol.

[185] ’t Hart BA, Elferink DG, Drijfhout JW, Storm G, van Blooijs L, Bontrop RE, de Vries RR (1997). Liposome-mediated peptide loading of MHC-DR molecules in vivo. FEBS Lett.

[186] Banchereau J, Klechevsky E, Schmitt N, Morita R, Palucka K, Ueno H (2009). Harnessing human dendritic cell subsets to design novel vaccines. Ann NY Acad Sci.

[187] Anderton SM, Manickasingham SP, Burkhart C, Luckcuck TA, Holland SJ, Lamont AG, Wraith DC (1998). Fine specificity of the myelin-reactive T cell repertoire: implications for TCR antagonism in autoimmunity. J Immunol.

[188] Bielekova B, Goodwin B, Richert N, Cortese I, Kondo T, Afshar G, Gran B, Eaton J, Antel J, Frank JA, McFarland HF (2000). Encephalitogenic potential of the myelin basic protein peptide (amino acids 83–99) in multiple sclerosis: results of a phase II clinical trial with an altered peptide ligand. Nat Med.

[189] Anderton SM (2001). Peptide-based immunotherapy of autoimmunity: a path of puzzles, paradoxes and possibilities. Immunology.

[190] Kappos L, Comi G, Panitch H, Oger J, Antel J, Conlon P, Steinman L (2000). Induction of a non-encephalitogenic type 2 T helper-cell autoimmune response in multiple sclerosis after administration of an altered peptide ligand in a placebo-controlled, randomized phase II trial. The Altered Peptide Ligand in Relapsing MS Study Group. Nat Med.

[191] Saraiva M, Christensen JR, Veldhoen M, Murphy TL, Murphy KM, O’Garra A (2009). Interleukin-10 production by Th1 cells requires interleukin-12-induced STAT4 transcription factor and ERK MAP kinase activation by high antigen dose. Immunity.

[192] Rogers PR, Huston G, Swain SL (1998). High antigen density and IL-2 are required for generation of CD4 effectors secreting Th1 rather than Th0 cytokines. J Immunol.

[193] Pfeiffer C, Murray J, Madri J, Bottomly K (1991). Selective activation of Th1- and Th2-like cells in vivo – response to human collagen IV. Immunol Rev.

[194] Tian DH, Perera CJ, Apostolopoulos V, Moalem-Taylor G (2013). Effects of vaccination with altered peptide ligand on chronic pain in experimental autoimmune encephalomyelitis, an animal model of multiple sclerosis. Front Neurol.

[195] Perera CJ, Duffy SS, Lees JG, Kim CF, Cameron B, Apostolopoulos V, Moalem-Taylor G (2015). Active immunization with myelin-derived altered peptide ligand reduces mechanical pain hypersensitivity following peripheral nerve injury. J Neuroinflamm.

[196] Cortes LM, Avichezer D, Silver PB, Luger D, Mattapallil MJ, Chan CC, Caspi RR (2008). Inhibitory peptide analogs derived from a major uveitogenic epitope protect from antiretinal autoimmunity by inducing type 2 and regulatory T cells. J Leukoc Biol.

[197] Lau LL, Jamieson BD, Somasundaram T, Ahmed R (1994). Cytotoxic T-cell memory without antigen. Nature.

[198] Norman PS, Nicodemus CF, Creticos PS (1997). Clinical and immunologic effects of component peptides in Allervax cat. Int Arch Allergy Immunol.

[199] Haselden BM, Kay AB, Larche M (1999). Immunoglobulin E-independent major histocompatibility complex-restricted T cell peptide epitope-induced late asthmatic reactions. J Exp Med.

[200] Oldfield WL, Kay AB, Larche M (2001). Allergen-derived T cell peptide-induced late asthmatic reactions precede the induction of antigen-specific hyporesponsiveness in atopic allergic asthmatic subjects. J Immunol.

[201] Oldfield WL, Larche M, Kay AB (2002). Effect of T-cell peptides derived from Fel d 1 on allergic reactions and cytokine production in patients sensitive to cats: a randomised controlled trial. Lancet.

[202] Alexander C, Ying S, A BK, Larche M (2005). Fel d 1-derived T cell peptide therapy induces recruitment of CD4^+^CD25^+^; CD4+ interferon-γ^+^ T helper type 1 cells to sites of allergen-induced late-phase skin reactions in catallergic subjects. Clin Exp Allergy.

[203] Alexander C, Tarzi M, Larche M, Kay AB (2005). The effect of Fel d 1-derived T-cell peptides on upper and lower airway outcome measurements in cat-allergic subjects. Allergy.

[204] Creticos PS, Hebert J, Philip G, The Allervax Ragweed Study Group (1997). Efficacy of Allervax ragweed in the treatment of ragweed-induced allergy. J Allergy Clin Immunol.

[205] Muller U, Akdis CA, Fricker M, Akdis M, Blesken T, Bettens F, Blaser K (1998). Successful immunotherapy with T-cell epitope peptides of bee venom phospholipase A2 induces specific T-cell anergy in patients allergic to bee venom. J Allergy Clin Immunol.

[206] Fellrath JM, Kettner A, Dufour N, Frigerio C, Schneeberger D, Leimgruber A, Corradin G, Spertini F (2003). Allergen-specific T-cell tolerance induction with allergen-derived long synthetic peptides: results of a phase I trial. J Allergy Clin Immunol.

[207] Tarzi M, Klunker S, Texier C, Verhoef A, Stapel SO, Akdis CA, Maillere B, Kay AB, Larche M (2006). Induction of interleukin-10 and suppressor of cytokine signalling-3 gene expression following peptide immunotherapy. Clin Exp Allergy.

[208] Texier C, Pouvelle S, Busson M, Herve M, Charron D, Menez A, Maillere B (2000). HLA-DR restricted peptide candidates for bee venom immunotherapy. J Immunol.

[209] Crowe PD, Qin Y, Conlon PJ, Antel JP (2000). NBI-5788, an altered MBP83–99 peptide, induces a T-helper 2-like immune response in multiple sclerosis patients. Ann Neurol.

[210] Kinnunen T, Jutila K, Kwok WW, Rytkonen-Nissinen M, Immonen A, Saarelainen S, Narvanen A, Taivainen A, Virtanen T (2007). Potential of an altered peptide ligand of lipocalin allergen Bos d 2 for peptide immunotherapy. J Allergy Clin Immunol.

